# Biochemical and structural characterization of tomato polyphenol oxidases provide novel insights into their substrate specificity

**DOI:** 10.1038/s41598-019-39687-0

**Published:** 2019-03-11

**Authors:** Ioannis Kampatsikas, Aleksandar Bijelic, Annette Rompel

**Affiliations:** Universität Wien, Fakultät für Chemie, Institut für Biophysikalische Chemie, Wien, Austria

## Abstract

Polyphenol oxidases (PPOs) contain the structurally similar enzymes tyrosinases (TYRs) and catechol oxidases (COs). Two cDNAs encoding pro-PPOs from tomato (*Solanum lycopersicum*) were cloned and heterologously expressed in *Escherichia coli*. The two pro-PPOs (*Sl*PPO1-2) differ remarkably in their activity as *Sl*PPO1 reacts with the monophenols tyramine (k_cat_ = 7.94 s^−1^) and phloretin (k_cat_ = 2.42 s^−1^) and was thus characterized as TYR, whereas *Sl*PPO2 accepts only diphenolic substrates like dopamine (k_cat_ = 1.99 s^−1^) and caffeic acid (k_cat_ = 20.33 s^−1^) rendering this enzyme a CO. This study, for the first time, characterizes a plant TYR and CO originating from the same organism. Moreover, X-ray structure analysis of the latent holo- and apo-*Sl*PPO1 (PDB: 6HQI and 6HQJ) reveals an unprecedented high flexibility of the gatekeeper residue phenylalanine (Phe270). Docking studies showed that depending on its orientation the gatekeeper residue could either stabilize and correctly position incoming substrates or hinder their entrance into the active site. Furthermore, phloretin, a substrate of *SI*PPO1 (K_m_ = 0.11 mM), is able to approach the active centre of *Sl*PPO1 with both phenolic rings. Kinetic and structural results indicate that phloretin could act as a natural substrate and connote the participation of PPOs in flavonoid-biosynthesis.

## Introduction

Polyphenol oxidases (PPOs) are metalloenzymes that contain a type-III copper centre and are omnipresent among bacteria^1^, fungi^2–6^, archaea^7^, plants^8–11^, insects^12,13^ and animals^14,15^. The PPO family is composed of three different types of enzymes, tyrosinases (TYRs)^16^, catechol oxidases (COs)^17^ and aurone synthases (AUSs)^18^. TYRs catalyse the *ortho*-hydroxylation of monophenols (monophenolase activity, EC 1.14.18.1) and the subsequent two electron oxidation of the resulting *o*-diphenols to the corresponding *o*-quinones (diphenolase activity, EC 1.10.3.1), whereas COs are only capable of catalysing the latter diphenolase reaction^19,20^. On the other hand, AUS represents a new class of PPOs that is functionally placed in between TYRs and COs and plays a significant role in the formation of aurones^18,21–23^. The quinones produced by PPOs are highly reactive and form *via* non-enzymatic reactions complex polymers known as melanins^24^. The occurrence of these dark brownish substances reduces the quality of many processed or fresh fruits and vegetables lowering their disposal value significantly^25^. However, melanins are highly important substances for plants as they participate in wound healing reactions and in the immune response against microorganisms and plant-pests^26–29^.

Plant PPOs are *in vivo* typically expressed as 55–65 kDa latent enzymes consisting of a N-terminal (40–45 kDa) and a C-terminal domain (15–20 kDa)^9,30^. The former domain represents the main domain as it harbours the (di-copper) active centre, whereas the latter domain is, among others, responsible for the enzyme’s latency by shielding its active site from substrates. The enzyme is activated by a so far unknown proteolytic process that separates the main- from the C-terminal domain. However, the latent enzyme can also be activated *in vitro* by proteases (e.g. trypsin, proteinase K)^5,31^, acidic or basic pH^32^, fatty acids^33^, and detergents (e.g. sodium dodecyl sulfate, SDS)^5,31,34,35^. In this context, the use of SDS seems not only to be the most commonly applied activation method but also the most accurate one as the SDS-mediated activation leads to activity rates that resemble very much those of the active enzyme^31^. PPOs exhibit three different oxidation states named *deoxy*-, *met*-, and *oxy*-form. Type-III copper enzymes usually exist in their resting state, i.e. *met*-form, which is able to react with diphenolic substrates but is incapable to accept monophenols. The reaction of the *met*-form with a diphenol reduces the copper ions (Cu^II^ → Cu^I^) and converts the enzyme into its inactive *deoxy*-from, which however can rapidly bind dioxygen in order to form the active *oxy*-form^36^.

*S. lycopersicum* contains six genes that encode different PPOs^37^. The six genes have been classified into three different classes according to Restriction Fragment Length Polymorphism (RFLP) and were grouped within separate clusters. *Sl*PPO-A (Q08303) and *Sl*PPO-E (Q08307), which correspond to the herein described isoenzymes *Sl*PPO1 (LR025217) and *Sl*PPO2 (LS999938), belong to different clusters and are thus supposed to exhibit different characteristics with respect to their membrane association in the thylakoid lumen. *Sl*PPO-A exhibits hydrophobic characteristics and is, therefore, able to interact with membranes, whereas *Sl*PPO-E is proposed to be more soluble^37^. In tomato plants, PPOs are involved in the defence mechanism as it was shown that the suppression of PPOs increases the plant’s susceptibility toward diseases^38^, whereas PPO overexpression enhances its resistance towards microorganisms like *Pseudomonas syringae*^39^. Additionally, tomato PPO activity has also been associated with resistance against insects like the common cutworm (*Spodoptera litura*)^28^. However, the specific substrates of tomato PPOs, which influence the defence mechanism of *S. lycopersicum,* are still unknown.

Here we show that the isoenzymes *Sl*PPO1-2 do significantly differ in their activity behaviour and pH stability, despite their sequences sharing 71.7% identity (Fig. S1). Based on kinetic results, *Sl*PPO1 was identified as TYR, whereas *Sl*PPO2 was classified as CO. This is the first time that a plant TYR and a CO originating from the same organism (*S. lycopersicum*) have been identified and biochemically characterized. The results demonstrate the significance for plant PPOs to harbour multiple PPO genes within their genome as the PPOs exhibit different substrate preferences, which indicates that they fulfil different physiological roles. The crystal structures of both the apo- and holo-form of *Sl*PPO1 were solved, which resembles those of other already published PPOs^16,17^. However, the gatekeeper residue (Phe270) of *Sl*PPO1 exhibits an unusually high flexibility. Docking studies applying the crystal structure of *Sl*PPO1 suggests that the gatekeeper residue might exhibit dual-functionality by selectively blocking or stabilizing substrate candidates depending on their structure. Furthermore, both kinetic and structural results indicate that the natural phenol phloretin possesses a high specificity for *Sl*PPO1 proposing that some PPOs could accept flavonoids as their natural substrates and thus could be involved in the secondary metabolite pathway^21^.

## Results and Discussion

### Heterologous expression and purification of *Sl*PPO1 and *Sl*PPO2

Complementary DNA was synthesized and used for the amplification of the two genes. Degenerated primers were designed (Table S1) in order to comprise any of the six genes encoding for PPOs in *S. lycopersicum*. Two genes were successfully cloned from young tomato leaves (1–2 cm) encoding for *Sl*PPO1 and *Sl*PPO2. *Sl*PPO1 (506 amino acids) and *Sl*PPO2 (500 amino acids) are isoenzymes of the published genes encoding *Sl*PPO-A (89.9% similarity) and *Sl*PPO-E (99.6% similarity)^37^, respectively. The sequence similarity between *Sl*PPO1 and *Sl*PPO-A is relatively low due to a natural stop codon, which is located within the complete sequence of *Sl*PPO-A, truncating the resulting sequence of *Sl*PPO1 by 37 amino acids in comparison to the published one of *Sl*PPO-A. The two isoenzymes were heterologously overexpressed in *E. coli* by a method that is similar to the expression protocol described elsewhere^31^. Different expression temperatures were examined revealing that the overexpression of *Sl*PPO1 was most efficient at a temperature of 25 °C, whereas the highest expression rate for *Sl*PPO2 was obtained at a temperature of 20 °C. The expression yields of the two isoenzymes differed remarkably, whereby the expression of *Sl*PPO1 was the most robust one yielding 80 mg of pure latent protein per litre of bacterial culture after the last purification step, whereas only 2 mg per litre of culture was obtained in the case of *Sl*PPO2. The final purity of both enzymes was >95% as judged by SDS-PAGE (Fig. S2).

### Molecular mass determination of *Sl*PPO1 and *Sl*PPO2

**Figure 1:**
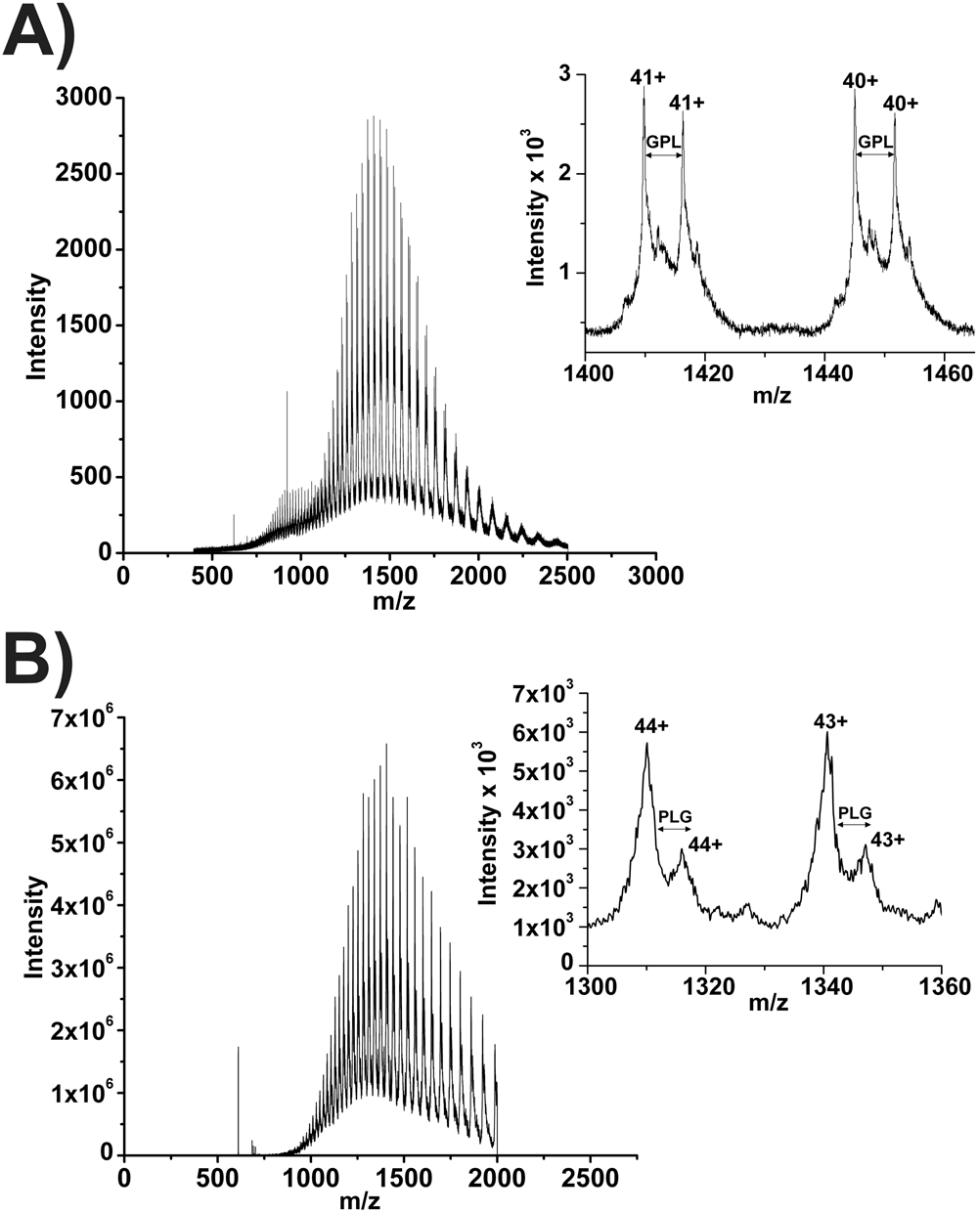
Mass spectra of the latent *Sl*PPO1 and *Sl*PPO2. Entire mass spectra of the acidified samples of (**A**) purified recombinant latent *Sl*PPO1 and (**B**) *Sl*PPO2. Insets show a magnified view of charge states.

The putative masses of *Sl*PPO1 and *Sl*PPO2 were calculated considering the presence of the two conserved disulphide bonds (−4.032 Da) and one thioether bridge (−2.016 Da). Based on this, the two proteins are supposed to have the theoretical masses (−6H) given in Table 1. ESI-MS yielded two masses for each *Sl*PPO enzyme (Fig. 1). The first mass (58027.60 ± 0.78) of *Sl*PPO1 matches the calculated mass of 58026.92 for the complete sequence of the latent *Sl*PPO1 including the two disulphide bonds and the thioether bridge. The second mass (57760.26 ± 0.51) indicates a proteolytic cleavage between a leucine (Leu3) and a glycine (Gly4), which are located within the remaining part of the expression vector (GPL|GSPEFP) after which the N-terminus of the recombinant enzyme starts. The removal of the first three N-terminal amino acids from the vector-derived region has also been observed before for PPO4 from *Agaricus bisporous*^5^. The first mass (57870.19 ± 0.66 Da) of latent *Sl*PPO2 indicates a proteolytic cleavage between the first glycine (Gly1) and proline (Pro2) residue of the remaining vector (G|PLGSPEFP). The second mass (57603.45 ± 0.98) corresponds to a cleavage between the second glycine (Gly4) and the adjacent serine (Ser5) of the expression vector (GPLG|SPEFP). Both masses of *Sl*PPO2 contain the two disulphide bonds and the thioether bridge.

**Table 1:** Masses of *Sl*PPO1-2 determined by mass spectrometry.

Enzyme	M (−6H) calculated Da	M (measured) Da	Δ/Da
*Sl*PPO1	58026.92	58027.60	+0.68 (−6H/measured)
*Sl*PPO1	58026.92	57760.26 ^(−GPL)^	−266.66 (−6H/measured)
*Sl*PPO2	57927.77	57870.19 ^(−G)^	−57.58 (−6H/measured)
*Sl*PPO2	57927.77	57603.45 ^(−GPLG)^	−324.32 (−6H/measured)

### Thermal shift assay of *Sl*PPO1 and *Sl*PPO2

**Figure 2:**
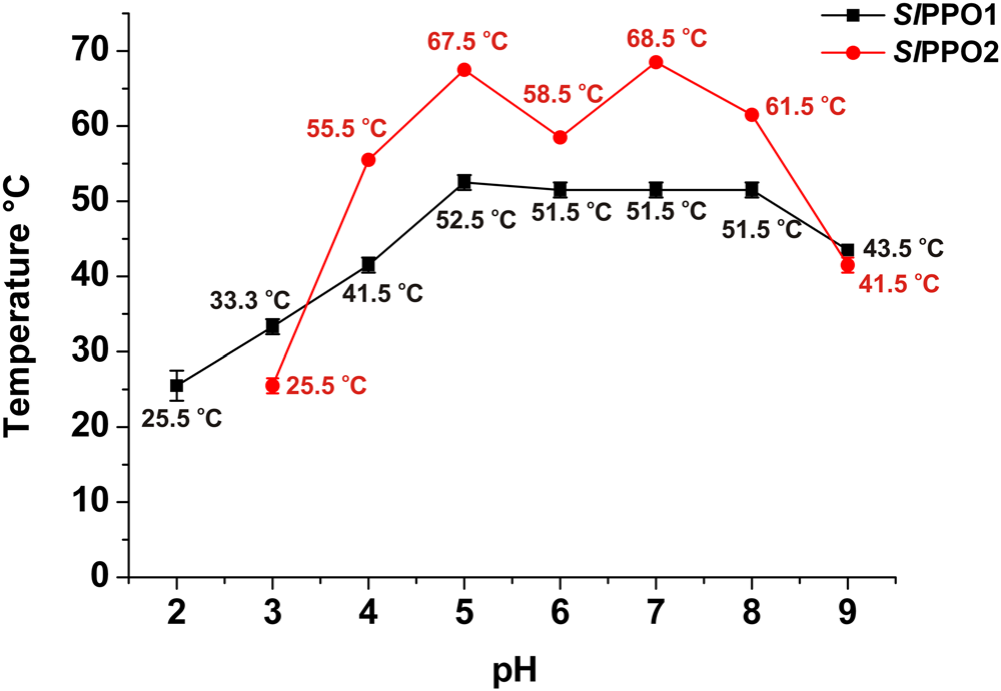
Thermal shift assay of *Sl*PPO1 and *Sl*PPO2 within the pH range of 2–9. The determined melting points are plotted against the respective pH values.

The stability of the purified proteins *Sl*PPO1 and *Sl*PPO2 were examined at different pH values (pH 2–9) by measuring their melting points using the thermal shift assay. The isoenzymes exhibited different denaturation points depending on the pH value indicating the importance of the pH for the stability of PPOs (Fig. 2). *Sl*PPO1 is most stable at pH 5–8 as it exhibits its highest melting point temperatures (51.5–52.5 °C) within this pH region. The stability of *Sl*PPO1 increases slowly from pH 2 to 4 before reaching its maximum stability at pH 5 to 8, which decreases again at pH 9. On the other hand, the shape of the stability curve of *Sl*PPO2 is more definite than that of *Sl*PPO1. The enzyme is unstable at pH 2 (no melting point was determined), however, the stability increases rapidly from pH 3 to 4 from a melting point of 25.5 to 55.5 °C, which means an incensement of 30 °C. *Sl*PPO2 has two peak melting points, 67.5 °C at pH 5 and 68.5 °C at pH 7. Similar to *Sl*PPO1, the stability of *Sl*PPO2 starts dropping at pH 9. In general, *Sl*PPO2 seems to be more stable than *Sl*PPO1 within the pH range of 4 to 8. The obtained stability information was used to derive and select appropriate pH values for the storing and crystallization buffers for each enzyme. Thus, *Sl*PPO1 and *Sl*PPO2 were stored at 50 mM Tris-HCl, pH 7.0. Since the probability of crystal formation increases with the stability of a protein, *Sl*PPO1 was crystallized within the pH range from 6.0 to 7.0. Indeed, the best crystals were obtained at pH 6.8 demonstrating the benefit of the thermal shift assay. Similarly, the best crystals of *Sl*PPO2 appeared within the pH range of 7.0 to 7.5, however, they did not diffract sufficiently (Fig. S3).

### Substrate specificity of *Sl*PPO1 and *Sl*PPO2

Substrate specificity for *Sl*PPO1 and *Sl*PPO2 was investigated by kinetic and substrate acceptance assays. Kinetic data of *Sl*PPO1 and *Sl*PPO2 were determined for four substrates, two monophenols (tyramine and phloretin) and two diphenols (dopamine and caffeic acid) (Table 2)^40^. Substrate acceptance assays were additionally performed to quickly check the acceptance of further mono- (tyrosine, tyrosol, (±)-octopamine, phenol, acetaminophen) (Fig. S4) and diphenols (catechol, 4-methylcatechol, 4-tertbutylcatechol, *L*-3,4-dihydroxyphenylalanine (*L*-DOPA), 3,4-dihydroxyphenylacetic acid (DOPAC), chlorogenic acid and butein) (Fig. S5). Testing different substrates provide valuable information on the substrate preferences of each isoenzyme.

**Table 2:** Kinetic parameters of *Sl*PPO1 and *Sl*PPO2 (activated with SDS).

Enzyme	Substrate	λ_max_ [nm]	ε_max_ [M^−1^cm^−1^]	K_m_ [mM]	k_cat_ [s^−1^]	k_cat_/K_m_ [s^−1^ mM^−1^]
*Sl*PPO1	tyramine	480	3300^52^	0.69 ± 0.12	7.94 ± 0.48	11.50 ± 2.12
	phloretin	455	11715^(this work)^	0.11 ± 0.01	2.42 ± 0.11	22.00 ± 2.24
	dopamine	480	3300^52^	0.67 ± 0.13	13.48 ± 1.15	20.12 ± 4.26
	caffeic acid	495	2062^52^	0.72 ± 0.04	11.90 ± 0.76	16.53 ± 1.40
*Sl*PPO2	tyramine	480	3300^52^	no activity	no activity	no activity
	phloretin	455	11715^(this work)^	no activity	no activity	no activity
	dopamine	480	3300^52^	5.82 ± 1.36	1.99 ± 0.30	0.34 ± 0.09
	caffeic acid	495	2062^52^	4.85 ± 2.25	20.33 ± 6.02	4.19 ± 2.30

In the case of monophenolase activity, *Sl*PPO1 shows high specificity for the monophenolic substrate phloretin (K_m_ = 0.11 mM), which is remarkably higher than that for the sterically smaller substrate tyramine (K_m_ = 0.69 mM). However, tyramine (k_cat_ = 7.94 s^−1^) is converted faster than the sterically demanding phloretin (k_cat_ = 2.42 s^−1^) indicating that the specificity does not correlate with the activity rate. Substrate acceptance assays with monophenolic substrates showed that the activity of *Sl*PPO1 with tyramine and phloretin was fast as the appearance of the chromophoric dyes appeared immediately in comparison to tyrosol, phenol and acetaminophen, where the chromophores appeared later but still earlier than in the case of tyrosine and (±)-octopamine (Fig. S6). On the other hand, *Sl*PPO2 was unable to react with any of the investigated monophenols (Fig. S6).

Regarding diphenolic substrates, both enzymes were active. The kinetic data show that *Sl*PPO1 exhibits similar reaction rates on both dopamine and caffeic acid (k_cat_ = 13.48 s^−1^ and 11.90 s^−1^, respectively). The specificity, according to K_m_, for dopamine (K_m_ = 0.67 mM) and caffeic acid (K_m_ = 0.72 mM) was also similar. In contrast to *Sl*PPO1, the affinity of *Sl*PPO2 towards dopamine (K_m_ = 5.82 mM) and caffeic acid (K_m_ = 4.85 mM) is relatively low indicating that these diphenols do not represent specific substrates for *Sl*PPO2. However, *Sl*PPO2 exhibits an unexpectedly high activity rate for caffeic acid (k_cat_ = 20.33 s^−1^), which is superior to that of *Sl*PPO1 (k_cat_ = 11.90 s^−1^) (Table 2). Substrate acceptance assays examining the catalytic reaction of *Sl*PPO1 and *Sl*PPO2 with different diphenolic substrates indicated that *Sl*PPO1 reacts with most of the substrates very fast, whereas *Sl*PPO2 shows a clear preference towards caffeic acid being either weakly or not active at all on the remaining diphenols (Fig. S6). The results unambiguously classify *Sl*PPO1 as TYR and *Sl*PPO2 as CO.

### Formation of the *oxy*-form in *Sl*PPO1 and *Sl*PPO2

**Figure 3:**
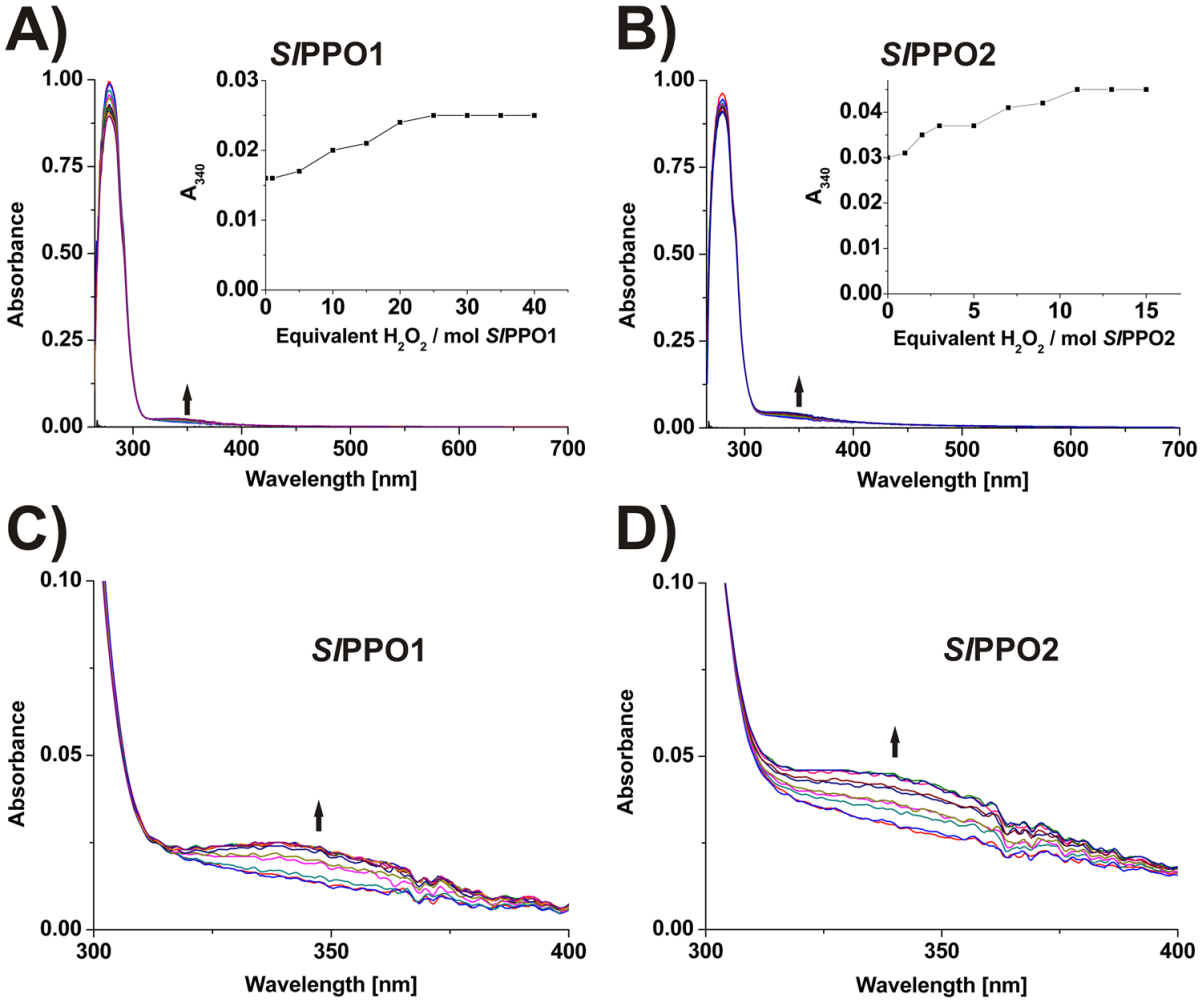
UV/Vis spectra of *Sl*PPO1 and *Sl*PPO2 after treatment with H_2_O_2_. (**A**) Spectra of *Sl*PPO1 after treatment with H_2_O_2_. Inset shows the absorption at 340 nm vs. equivalents of H_2_O_2_. (**B**) Spectra of *Sl*PPO2 after treatment with H_2_O_2_. Inset shows the absorption at 340 nm vs. equivalents of H_2_O_2_. (**C**) Zoom at 300–400 nm of the spectra of *Sl*PPO1 after treatment with H_2_O_2_. (**D**) Zoom at 300–400 nm of the spectra of *Sl*PPO2 after treatment with H_2_O_2_.

*Sl*PPO1 and *Sl*PPO2 were spectrophotometrically examined using H_2_O_2_. Addition of H_2_O_2_ to *Sl*PPO1 and *Sl*PPO2 leads to a new absorption band around 345 nm which is characteristic for the oxygen-induced *oxy*-form. Saturation of the band of *Sl*PPO1 at 340 nm with an extinction coefficient of ~1360 M^−1^ cm^−1^ per protein is reached at 25 equiv. of H_2_O_2_, while, the band of *Sl*PPO2 with an extinction coefficient of ~2570 M^−1^ cm^−1^ per protein is saturated at 11 equiv. of H_2_O_2_ (Fig. 3). The formation of the *oxy*-form by H_2_O_2_ has previously been shown for other PPOs^10,41^, however, this is the first study investigating the *oxy*-form formation of recombinantly expressed PPOs.

### Crystallization of the latent apo- and holo-form of *Sl*PPO1

After purification, both *Sl*PPO1 and *Sl*PPO2 were subjected to crystallization, however, X-ray diffraction data were only obtained for *Sl*PPO1 as the quality of the *Sl*PPO2 crystals was insufficient for data collection. Later crystal packing analysis revealed that, despite sharing 71.7% sequence identity (Fig. S1), both isoenzymes differ especially in their surface exposed amino acid sequence explaining the failed attempt to obtain high quality crystals of *Sl*PPO2. *Sl*PPO1 was crystallized as latent apo- and holo-enzyme (without and with copper).

### Crystal structure of latent holo-*Sl*PPO1

**Figure 4:**
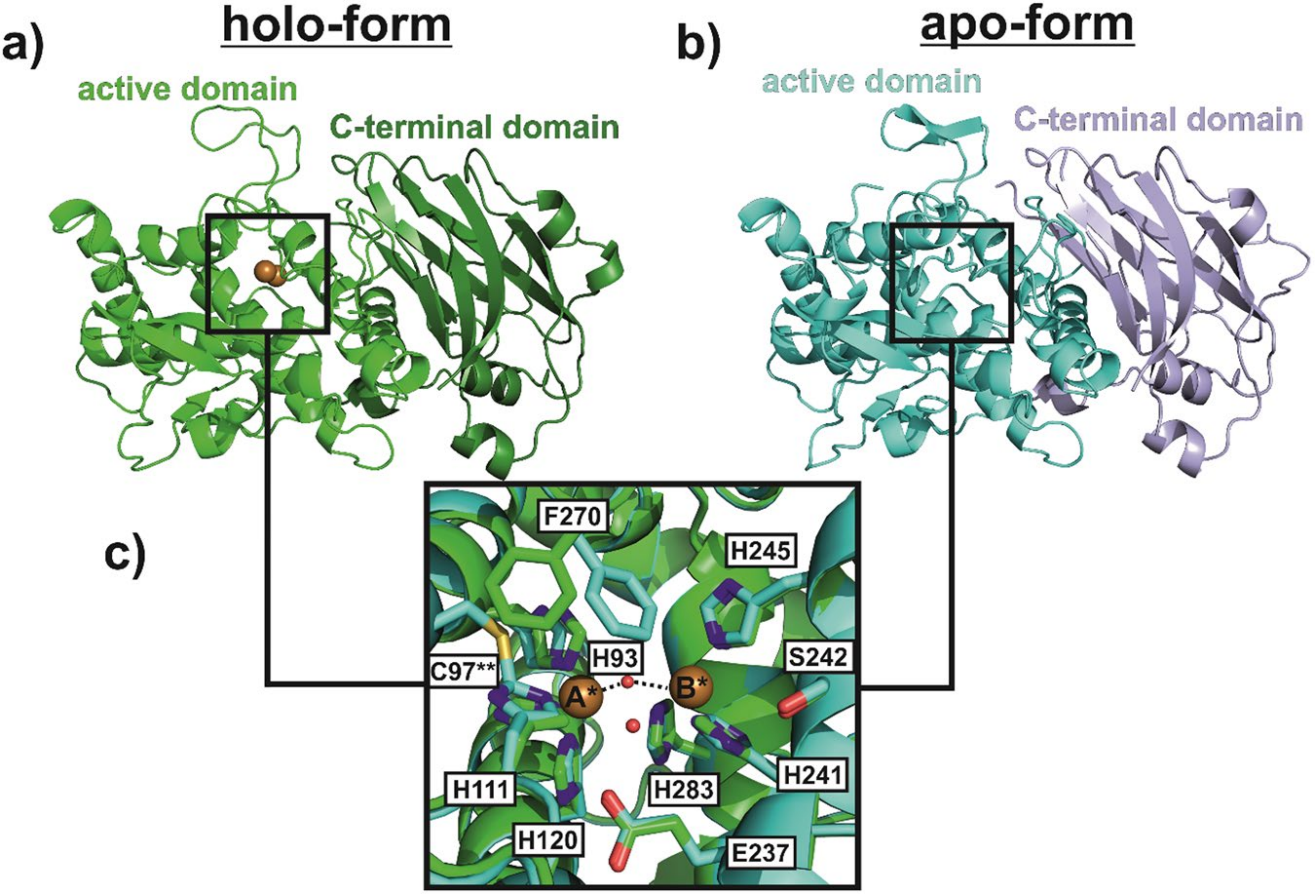
Overall and active site structure of holo- and apo-*Sl*PPO1. (**a**) Overall structure of holo-*Sl*PPO1. (**b**) Overall structure of apo-*Sl*PPO1. (**c**) Superimposition of the active centre of holo- (green structure) and apo-*Sl*PPO1 (cyan structure). The copper ions bridging oxygen moiety is illustrated as small red sphere coordinating to both copper ions (indicated by black dashes), whereas the water molecule modelled in the apo-form is located beneath the bridging oxygen moiety (without black dashes). *Indicates that the copper ions are only present in the holo-form. **Indicates that the thioether bridge is only present in the apo-form. Please note the significant differences in the position of the gatekeeper residue (Phe270).

The X-ray structure analysis of the holo-form was determined at 1.85 Å resolution (PDB entry 6HQI). As expected the overall core structure and especially the active site region of holo-*Sl*PPO1 resembles those of other structurally known plant PPOs (e.g. tyrosinase from *Juglans regia*^10,16,42^, catechol oxidases from *Ipomoea batatas*^17^ and *Vitis vinifera*^43^ and aurone synthase from *Coreopsis grandiflora*)^18,21,22^. The active site region is composed of a typical four-α-helical bundle harboring the dicopper active centre, where each copper ion (CuA and CuB) is coordinated by three histidine residues (Fig. 4). The structure of the holo-form lacks a large part of the N-terminal domain (model starts at Ser35) leading to the absence of two highly conserved disulphide bonds (Cys11-Cys27 and Cys26-Cys94). The absence of this N-terminal part could be explained by an X-ray induced cleavage of the conserved disulphide bonds leading to the destabilization of this part and thus to a significant increase in its flexibility diminishing the interpretable amount of electron density for this part^44^. Another possible reason could be that the N-terminal domain was lost by degradation. A characteristic structural feature of some PPOs is the formation of the thioether bridge which is missing in *Sl*PPO1. This bond is supposed to be formed between the second CuA coordinating histidine (His111) and an adjacent cysteine (Cys97). However, the structure of *Sl*PPO1 lacks a large solvent exposed loop (Cys97-Leu117) on which the thioether bond forming Cys97 is located most probably due to the same reasons mentioned above for the N-terminal tail. The structure suffers from further gaps owing to solvent exposed loop regions exhibiting excessive conformational disorders (Pro223-Ser228 and Leu447-Thr459). Despite these gaps, especially that one between the first and second CuA coordinating histidines (His93 and His111), the electron density of the active site region indicates an intact dicopper centre. The two copper ions exhibit low occupancy values (CuA = 0.3 and CuB = 0.1) which might be the result of copper loss during the X-ray diffraction experiment^45^. The presence of an ‘oxygen moiety’ between the copper ions suggests that the enzyme was crystallized in its *met*-form with a CuA-CuB distance of 4.2 Å, which is in accordance to the *met*-forms of other structurally known plant PPOs (Fig. 4)^16,17^.

One striking difference in comparison to other plant PPO structures is the position of the gatekeeper residue, Phe270. This residue exhibits an unexpected low electron density indicating an unusually high conformational disorder (Figs. S7 and S8). Electron density of the main conformer of Phe270 starts to appear only at low contour levels (~0.6 σ) (Figs. S7 and S8). The position of Phe270 is significantly shifted in comparison to the gatekeeper residue position in other plant PPO structures further confirming its high flexibility, which might have an impact on the catalytic behaviour of *Sl*PPO1^16^. Another highly interesting structural feature of *Sl*PPO1 is the amino acid residue located at the position of the 1^st^ activity controller (residue following the first CuB coordinating histidine)^31^. In contrast to most TYRs, *Sl*PPO1 contains a serine residue (Ser240) instead of asparagine or aspartic acid at this position. This clearly contradicts the existing theory that an asparagine (or aspartic acid) is required at this position for TYR activity^46,47^. Besides *Sl*PPO1, different TYRs from apple do also possess other residues (alanine and glycine) than asparagine at this activity controller position^31,34^.

### Crystal structure of latent apo-*Sl*PPO1 and comparison with holo-*Sl*PPO1

The X-ray structure of the apo-form was determined at 1.80 Å resolution (PDB entry 6HQJ). The structure of apo-*Sl*PPO1 is, in general, the same as that of the holo-form (Fig. S7b,d). Despite the lack of copper ions, there is electron density present in the centre of the active site, which was modelled as a water molecule. The apo-structure suffers from similar imperfections as the holo-form. The N-terminus of the apo-form starts at Ala28 and thus lacks also the two conserved disulphide bonds. Furthermore, the structure exhibits gaps in the same loop regions as the holo-form (Thr225-Thr229 and Pro448-Thr459). However, there are also significant differences between the holo- and the apo-structure. The loop Cys97-Leu117, which is missing in holo-*Sl*PPO1, is present in the apo-form, leading to the presence of an intact thioether bridge (Fig. S9). Similar to the holo-structure, the gatekeeper residue Phe270 in the apo-structure exhibits hardly any electron density, which confirms the flexible nature of the gatekeeper residue in tomato PPOs (Figs. S7 and S8). In contrast to holo-*Sl*PPO1, the electron density of Phe270 in the apo-structure, which also starts to appear only at a low contour level (~0.6 σ), is located at a position similar to the gatekeeper residue position found in other plant PPOs. Comparison of the gatekeeper residue positions between the apo- and the holo-structure reveals a significant positional shift (Fig. S8), which is only possible owing to the absence of the thioether bridge in the holo-structure. The Phe270 residue of the holo-form would sterically interfere with a present thioether bridge (Fig. S9). This observation indicates that the thioether bridge might have stabilizing effects on the position of the gatekeeper residue in PPOs. In addition and in contrast to the holo-structure, the conserved water molecule, which is believed to deprotonate monophenolic substrates, is present in the apo-structure.

The superimposition of both the holo- and the apo-structure results in a (theoretically) complete and typical plant PPO structure as it would possess an intact dicopper active site, an intact thioether bridge and a conserved water molecule that is stabilized by the waterkeeper residue Glu237. To exclude the possibility that the absence of some structural features (i.e. thioether bridge and conserved water molecule) in the structure of holo-*Sl*PPO1 was just an exceptional case, we evaluated and analyzed collected data sets of further apo- and holo-*Sl*PPO1 crystals (Table S2). The results indicated that all apo-structures (based on two data sets) were identical, whereas the structures of the holo-enzymes (based on five data sets) differed slightly from one another. Two of the five holo-structures contained the conserved water molecule, whereas the remaining structural features (i.e. position of Phe270, copper content and the absence of the thioether bridge) were identical. This indicates that the conserved water molecule is not absolutely absent in holo-*Sl*PPO1, whereas the thioether bridge seems indeed to be missing. The reason for the absence of the Cys97 harboring loop in all holo-structures is not clear and it cannot be excluded that the loop underwent degradation. However, this does not explain thioether bond formation in all apo-forms, which were processed the same way as the holo-form. Thus, it remains unclear why the thioether bond was not formed in the holo-form and to what extent the X-ray data do reflect the situation in solution.

### Structural information on the substrate specificity/affinity of *Sl*PPO1 towards phloretin

**Figure 5:**
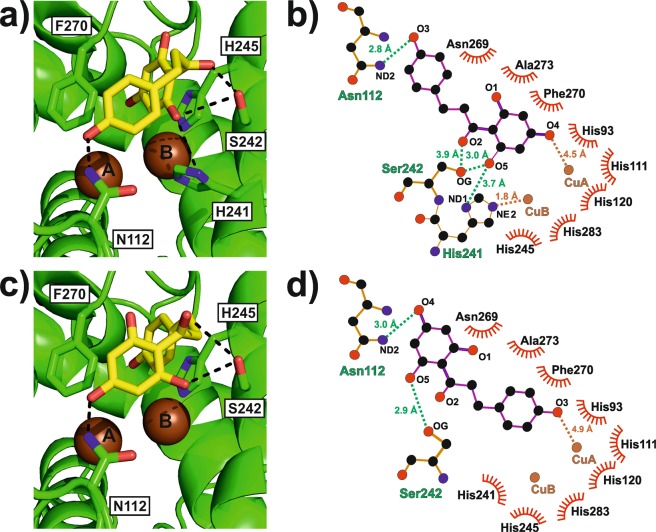
Binding of phloretin to *Sl*PPO1 according to molecular docking. (**a**) Binding pose 1 of phloretin within the active site of *Sl*PPO1. The molecule approaches CuA with its 2,4,6-trihydroxyphenyl ring. Phloretin is stabilized by three amino acids. Figure S11 shows the same figure from another perspective to provide a better view of the substrate’s conformation within the binding site. (**b**) Protein-ligand interaction plot of the *Sl*PPO1-phloretin complex (binding pose 1). (**c**) Binding pose 2 of phloretin within the active site of *Sl*PPO1. The molecule approaches CuA with its 4-hydroxyphenyl ring. The substrate interacts only with two amino acids. (**d**) Protein-ligand interaction plot of the *Sl*PPO1-phloretin complex (binding pose 2).

According to kinetic data, *Sl*PPO1 showed the highest affinity towards phloretin (K_m_ = 0.11 mM), whereas the affinity towards the remaining substrates (tyramine, caffeic acid and dopamine) was similar but clearly inferior to that of phloretin (see Table 2). To gain more information on the PPO-substrate interactions, molecular docking studies have been performed applying the crystal structure of holo-*Sl*PPO1 and all kinetically tested substrates. All computed docking poses were checked for their reasonableness by comparing them to the binding pose of tyrosine from the crystal structure of tyrosine-bound tyrosinase from *Bacillus megaterium* (*Bm*TYR, PDB entry 4P6R)^1^. Docking poses not matching the orientation of tyrosine in *Bm*TYR to a certain degree were flagged ‘unreasonable’. In all cases, the main driving force for ‘reasonable’ docking poses was the *π*-stacking system established between the aromatic ring of the gatekeeper residue Phe270, the substrate’s aromatic ring and the imidazole group of the CuB coordinating His245 (*π*-stacking system: Phe-substrate-His). Comparison of the docking poses of all *Sl*PPO1-substrate complexes revealed that phloretin exhibits binding poses that are stabilized much better than those of the other substrates owing to its bulky structure. Phloretin (3-(4-hydroxyphenyl)-1-(2,4,6-trihydroxyphenyl)propan-1-one) is able to approach the dicopper centre with both hydroxyphenyl groups, whereby the approach of the 2,4,6-trihydroxyphenyl ring is favoured. This pose enables more direct interactions between the substrate and the enzyme than in the binding scenario, where the 4-hydroxyphenyl ring is approaching the copper ions (Figs. 5, S10 and S11). The *para*-hydroxy group of the 2,4,6-trihydroxyphenyl moiety exhibits the lowest pK_a_ value and therefore it is most readily deprotonated, which is in accordance to the preferred docking pose. The pose with the 2,4,6-trihydroxyphenyl group approaching the copper ions (pose 1) is stabilized by three amino acids, the 1^st^ activity controller Ser242, the CuB coordinating His241 and Asn112. Ser242 hydrogen binds both the carbonyl and the *ortho*-positioned hydroxy group of the 2,4,6-trihydroxyphenyl moiety of phloretin (Fig. 5a,b). The same *ortho*-hydroxy group of phloretin forms a further hydrogen bond with the H-atom of the ND1 nitrogen atom of His241, which is not involved in the coordination of CuA. Asn112 exhibits a hydrogen bond with the hydroxy group of the 4-hydroxyphenyl ring, which is pointing away from the active site (Fig. 5a,b). In contrast, when the 4-hydroxyphenyl ring is approaching the active site (pose 2), phloretin interacts only with two amino acids, Asn112 and Ser242. Asn112 hydrogen binds the *para*-hydroxyl group of the 2,4,6-trihydroxyphenyl ring, whereas the activity controller is involved in an H-bond with an *ortho*-positioned hydroxy group of the same ring (Fig. 5c,d). The docking results of the remaining substrates revealed that they interact only with the activity controller Ser242 *via* the functional group at their tail (i.e. the amine group in the case of tyramine and dopamine, and the carboxyl group in the case of caffeic acid). These findings are also reflected by the computed affinity scores of the docking software as the best binding pose of phloretin exhibited the highest affinity −7.2 kcal/mol (Table S3), however, these docking scores do not represent reliable estimates for binding energies. Moreover, the analysis of all docking poses of each substrate (10 poses were calculated for each protonation state, see Methods) revealed that phloretin was the substrate that exhibited by far the largest number of ‘reasonable’ docking poses. About 75% of the computed phloretin poses were ‘reasonable’, whereas in the case of the remaining substrates only 5–17% of the docking poses represented ‘reasonable’ binding poses.

The same experiment was also conducted with *Sl*PPO2 by using a homology model of this enzyme based on the structure of *Sl*PPO1. However, the docking experiment failed to explain the absence of monophenolase activity in PPO2 as both enzymes led to similar binding poses owing to the almost identical architecture of their active sites. Nevertheless, in the case of phloretin, the docking experiment provided some valuable structural information on the binding discrepancy between PPO1 and PPO2, which are shown in the Supporting Information (Fig. S10).

### Molecular docking confirms the flexibility of the gatekeeper residue and suggests dual-functionality for this residue

The gatekeeper residue Phe270 plays an important role during substrate binding as it is supposed to stabilize (together with His245) the substrate *via π*-stacking. According to a previous theory, a bulky residue at this position was believed to act as an active site blocker by preventing the access of monophenolic substrates into the active site of COs^48^. This theory was contradicted by the crystal structure of walnut TYR (*jr*PPO1) that possesses phenylalanine as gatekeeper residue^16^. However, during the here presented docking study a series of substrate poses were calculated, where the Phe-gatekeeper residue exhibited positions that indeed suggest a blocking role for this residue. The docking study revealed that the gatekeeper residue, owing to its high flexibility, can interact with small substrates (lacking a long chain at the tail of their structure or an additional ring systems) in such a way that prevents them from accessing the active site. In these cases, Phe270 is positioned directly above CuA and exhibits unfavorable *π*-stacking with small substrates (tyramine, dopamine and to a lesser extend caffeic acid) repelling them from the dicopper centre. Due to these unfavorable *π*-stacking interactions, the substrates are hardly able to bypass the gatekeeper residue. This situation is additionally complicated by the activity controller Ser242 as it forms H-bonds with the substrates, which further stabilize and thus lock them in these unfavorable poses (Fig. S12). 80–95% of the docking poses of tyramine, dopamine and caffeic acid were flagged as ‘unreasonable’, whereby in the majority of these ‘unreasonable’ poses the gatekeeper residue blocked the substrate from accessing the active site (substrates were located outside the dicopper site, Fig. S12). Regarding the sterically demanding substrate phloretin, only ~25% of the calculated poses showed substrate binding outside the binding cleft. Thus, in the case of incoming small substrates (which might be unable to sufficiently interact with surrounding amino acids owing to their small size) *π*-stacking with the gatekeeper residue might represent the dominating interaction on their way to the dicopper centre. Thus, the position/flexibility of the gatekeeper residue could decide, whether the substrate will be orientated correctly within the binding site or blocked away from it. However, the factors determining the degree of the flexibility and thus the orientation of the gatekeeper residue and its effect on the enzyme’s substrate preference are not known. The docking results suggest a dual-functionality for the Phe-gatekeeper-residue, which on the one hand is able to stabilize the correct orientation of some substrates within the active site and on the other hand can also block the entrance of other substrates into the active site to some extent.

## Conclusions

PPOs from *S. lycopersicum* (*Sl*PPO1-2) were recombinantly produced, purified and kinetically and biochemically characterized, whereby one PPO (*Sl*PPO1) was also successfully crystallized in its apo- and holo-form. The two isoenzymes, sharing a high sequence similarity of 71.7%, exhibited significant differences in their substrate preferences classifying *Sl*PPO1 as TYR and *Sl*PPO2 as CO. One of the main hurdles in the field of PPOs is the identification of natural substrates. *Sl*PPO1 exhibits a high specificity towards the chalcone phloretin (as shown by kinetic and docking data), which might indicate that PPOs could accept flavonoids as their natural substrates and therefore might participate in the synthetic pathways of secondary metabolites^21^. Despite exhibiting a high binding affinity towards *Sl*PPO1 owing to its bulky structure and functional groups, the conversion rate for phloretin is significantly lower than that for the other tested substrates indicating that the hydroxylation process of phloretin (expressed as conversion rate) might be hampered by either its bulkiness or strong binding (=reduced flexibility within the binding site) or both.

The X-ray analysis of *Sl*PPO1 reveals that the bulky gatekeeper residue (Phe270) has an unprecedented high flexibility. Subsequent molecular docking did not only provide highly valuable insights into the binding event of different substrates but also confirmed the highly flexible nature of the gatekeeper residue. Based on the docking results, a dual-functionality for the gatekeeper residue is proposed as Phe270 did not only stabilize substrates *via π*-stacking during the simulations but was also able to shield the active site from approaching substrates by the same kind of interactions. Depending on the flexibility of the Phe-gatekeeper residue, i.e. on its position and orientation, and the structure of the substrate, the substrate-stabilizing effect of this residue might be more or less pronounced. However, the origin of this high flexibility, which never has been reported before for PPOs, and its total effect on the enzyme’s catalytic activity remain unknown.

According to the heterologous expression, *Sl*PPO1 is more soluble than *Sl*PPO2, which is in contradiction to their structural characteristics and expected interaction behaviour with membranes as only the former enzyme is supposed to be membrane associated^37^. PPOs are type-III copper enzymes exhibiting significant oxidation reactions in the majority of organisms. Plenty of PPO enzymes have been purified and biochemically characterized, however, only few have been produced purely and were crystallized in order to characterize their biochemical features accurately. The identification of natural substrates of PPOs represents a tough challenge in this field. However, *Sl*PPO1 exhibits a high specificity towards the chalcone phloretin, which might indicate that PPOs could accept flavonoids as natural substrates and therefore might participate in the synthetic pathways of secondary metabolites.

## Methods

All chemicals have been purchased from Sigma-Aldrich (Vienna, Austria) and Carl-Roth (Karlsruhe, Germany) and were at least of analytical grade.

### Plant material, cloning and sequencing of *Sl*PPO1 and *Sl*PPO2

Young healthy leaves from tomato plants (*S. lycopersicum*) were ground in liquid nitrogen, and the total RNA was isolated using the RNeasy Plant Mini Kit (Qiagen, Hilden, Germany) according to the manufacturer’s instructions. cDNA was synthesized using the SMARTer^®^ RACE cDNA Amplification Kit (Clontech, Saint-Germain-en-Laye, France) and an oligo d(T) 25 primer. Two pairs of degenerated primers (Table S1) were designed for at least six different PPOs, which have been placed in the genome of the *S. lycopersicum*^37^. Two different PPO genes were amplified from the cDNA template with Q5^®^ High-Fidelity DNA polymerase (NEB, Ipswich, England). PCR products were cloned into the pGEX-6P-1 expression vector (GE Healthcare, Freiburg, Germany) as follows: Once obtained, the amplified PPO genes were phosphorylated with T4 Polynucleotide kinase (NEB) and mixed with the pGEX-6P-1 vector that has been digested with the SmaI restriction enzyme (Thermo Fisher scientific, Massachusetts, USA) and dephosphorylated with Calf Intestinal Alkaline Phosphatase (NEB) to prevent religation of the linearized plasmid-DNA. The resulting mixtures were ligated with T4 DNA ligase (NEB) and transformed into chemically component *E. coli* TOP10 cells (Thermo Fisher scientific)^49^. The clones were sequenced externally by microsynth GmbH (Vienna, Austria).

### Heterologous expression and purification of recombinant *Sl*PPO1 and *Sl*PPO2

*Sl*PPO1 and *Sl*PPO2 genes were N-terminally fused with the GST-tag of the pGEX-6P-1 vector. The human rhinovirus 3C protease (HRV3C) recognition sequence (LEVLFQ|GP) was located between the two fusion partners enabling the controlled proteolytic dissociation of the two proteins. The two fusion genes (GST-*Sl*PPO1 and GST-*Sl*PPO2) were efficiently overexpressed using the synthetic tac promoter of the pGEX-6P-1 vector. *Escherichia coli* was grown in a modified 2xYT medium (1.6% tryptone-peptone, 1% yeast extract, 1% NaCl, 0.5% NH_4_Cl, 0.5% glycerol, 2 mM MgCl_2_, 1 mM CaCl_2_ at pH 7.5) supplemented with ampicillin (100 µg/ml). The expression batches were inoculated with saturated overnight cultures and grown at 37 °C under shaking for 4 hours until the OD_600_ reached a value between 0.6 and 0.8. Afterwards, the temperature of the *Sl*PPO1 containing approach was reduced to 25 °C, whereas that of the *Sl*PPO2 was reduced to 20 °C. The cultures were induced with 0.5 mM isopropyl β-D-1-thiogalactopyranoside and 0.5 mM CuSO_4_. The expression cultures remained at 25 °C and 20 °C under shaking for 24 and 48 hours, respectively. The cultures were then collected by centrifugation at 10000 × g for 25 minutes at 4 °C. Lysis of the cells was effectuated by the freeze-thaw technique using liquid nitrogen. The pellets were re-suspended in the lysis buffer (50 mM Tris-HCl pH 7.5, 200 mM NaCl, 1 mM EDTA and 50 mM sucrose). Lysozyme (0.5 g/l) and protease inhibitors (1 mM phenylmethylsulfonyl fluoride and 1 mM benzamidine) were added and the resulting suspensions were incubated for 45 minutes under shaking on ice. Subsequently, the solutions underwent five cycles of freezing in liquid nitrogen and thawing in a water bath at 25 °C. Eventually, 2 mM MgCl_2_ and 0.02 g/l DNaseI were added to the lysates, which were then incubated for 15 minutes at 100 rpm and 25 °C. The lysates were centrifuged at 10000 × g for 1 hour at 4 °C. The chromatographic purifications were carried out using an Äkta Purifier (GE Healthcare) placed in a refrigerator at 4 °C. The filtrated lysates were placed in a 50 ml injection loop and applied onto a prepacked 5 ml GSTrap FF column using 50 mM Tris-HCl pH 7.5 and 200 mM NaCl as the binding buffer. Following the trapping and flushing out of unbound proteins, the target proteins were eluted with 50 mM Tris-HCl pH 7.5, 200 mM NaCl and 15 mM reduced glutathione. Fractions containing the GST-fusion protein were pooled and concentrated using a Vivaspin ultrafiltration device with a 30 kDa molecular weight cut-off (VWR). The buffer was then exchanged to 50 mM Tris-HCl pH 7.0, 200 mM NaCl, 1 mM EDTA and the samples were mixed with GST-HRV3C, which were produced in-house^5^ at a mass ratio of 1:50 (protease: fusion protein). The proteolysis was carried out over 48 hours at 4 °C. The cleaved protein was then again applied onto a 5 ml GSTrap FF column, whereby the GST protein and the GST-tagged protease were still trapped by the column, while the latent PPOs passed through the column and were immediately eluted in the flowthrough. Subsequently, the two enzymes were applied to size exclusion chromatography (SEC) using a Superdex^®^ 200 increase 10/300 GL and the protein fractions of the latent *Sl*PPO1-2 were collected, concentrated and stored in 50 mM Tris-HCl pH 7.0. The protein concentrations were determined according to the Lambert-Beer law and their absorption at 280 nm using the extinction coefficient provided by ExPASy ProtParam^50,51^.

### Molecular mass determination by ESI-QTOF-MS and ESI-LTQ-Orbitrap-Velos

Electrospray Ionization Mass Spectrometry (ESI-MS) of *SI*PPO1 was performed on a nano electrospray ionisation–quadrupol and time-of-flight mass spectrometer (ESI-QTOF-MS, MaXis 4G UHR-TOF, Bruker) with a mass range of 50–20000 m/z applying the positive mode. Pure latent enzyme at a concentration of 10 g/l was used. The buffer was exchanged to 5 mM ammonium acetate (pH 7.0) and the enzyme solution was diluted to 1% (v/v) in 2% acetonitrile and 1‰ formic acid immediately before being applied to the mass spectrometer. Mass determination of *SI*PPO2 was performed by an ESI-LTQ-Orbitrap Velos (Thermo Fisher Scientific Bremen, Germany) with a mass range of 200–4000 m/z and a mass accuracy close to 3 ppm with external calibration. Prior to MS *SI*PPO2 solution was ultra-filtrated by centrifugation and the buffer was exchanged to 5 mM ammonium acetate (pH 7.0) and the protein solution was diluted 100 times in a mixture of 80% (v/v) acetonitrile and 0.1% (v/v) formic acid.

### Enzyme kinetics and activity assays

The activity was determined spectrophotometrically by detecting the appearance of the chromophoric quinones, which are produced by the reaction of the substrates (monophenols: tyramine and phloretin; diphenols: dopamine and caffeic acid) with the respective enzyme, in order to determine the kinetic parameters of latent *Sl*PPO1 and *Sl*PPO2. Absorption curves and spectra were recorded at 25 °C in a 96 well microplate applying a TECAN infinite M200 (Tecan). Kinetic measurements were performed using a total volume of 200 µl, containing 50 mM Tris-HCl buffer (pH 7.0), different molarities of substrates, different molarities of the enzyme and 1.5 mM SDS (for activation). Additionally, the acceptance of further monophenolic (tyrosine, tyrosol, (±)-octopamine, phenol and acetaminophen) and diphenolic substrates (catechol, 4-methylcatechol, 4-tert-butylcatechol (TBC), *L*-3,4-dihydroxyphenylalanine (*L*-DOPA), 3,4-dihydroxyphenylacetic acid (DOPAC), chlorogenic acid and butein) were determined for both enzymes by substrate acceptance assays. The molar absorption coefficients (ɛ_λmax_) of the formed chromophores of tyramine, dopamine and caffeic acid have already been reported in the literature (Table 2)^52^. The coefficient value for the monophenol phloretin was determined in 50 mM Tris buffer (pH 7.0) using the tyrosinase *Md*PPO1^31^. The molar extinction coefficient was then determined by linear regression at the appropriate wavelength (λ_max_) (Fig. S13). Spectra were taken routinely for each substrate on a Shimadzu UV-1800 spectrophotometer (Shimadzu Deutschland, Duisburg, Germany) using 1 ml solution at 25 °C.

### Thermal shift assay of *Sl*PPO1 and *Sl*PPO2

Thermal shift assay was conducted to measure the melting points of purified *Sl*PPO1 and *Sl*PPO2 at different pH values (pH range 2–9, in 1 pH unit increments) in order to determine the pH-dependent stability of each enzyme (pH 2–6 in 50 mM sodium citrate and pH 7–9 in 50 mM Tris-HCl buffer). The assay was performed in triplicates, using a 96-well PCR plate (Eppendorf AG, Hamburg) and a real-time PCR instrument (mastercycle^®^ ep-realplex Eppendorf). The total reaction volume of the solutions was 100 µl, which consisted of 7.5 µM pure enzyme, 4x SPYRO Orange (Sigma-Aldrich) and buffer to maintain the respective pH (pH 2–9). The plate was sealed with an optically clear film (Eppendorf). For the experiment, the plate was heated from 4 to 94 °C (in increments of 1 °C) in the PCR machine. The fluorescence changes were monitored simultaneously by measuring the fluorescence emission at 560 nm following excitation at 470 nm. The resulting melting points of each enzyme were then plotted against the respective pH value for stability analysis.

### Crystallization of the apo- and holo-form of *Sl*PPO1

Obtaining single crystals of sufficient quality was only achieved for holo- and apo-*Sl*PPO1 as crystals of *Sl*PPO2 did not diffract X-rays. The crystallization of *Sl*PPO1 was performed by applying the hanging drop vapour-diffusion technique using 15 well EasyXtal plates (Qiagen). Single crystals of *Sl*PPO1 were grown at 20 °C by mixing 1 µl of protein solution (10 mg ml^−1^) with 1 µl of the reservoir solution (50 mM sodium citrate pH 6.8, 13% w/v PEG 8000). Crystals usually appeared after 4 d. The apo-form was produced by the removal of the Cu ions from the dicopper centre. For this reason, *Sl*PPO1 (1 mg) was mixed with 200 mM ethylenediaminetetraacetic acid (EDTA) and 100 mM KCN at pH 8.0. The solution was incubated for 45 minutes and the buffer was exchanged to 50 mM Tris-HCl pH 7.0. This procedure was repeated three times and the final apo-*Sl*PPO1 was confirmed by activity assays as the enzyme was unable to react with any of the monophenolic or diphenolic substrates.

### Data collection, structure determination and refinement

The crystals of both apo- and holo-*Sl*PPO1 were harvested in nylon loops, soaked in a cryo-protectant solution (100 mM sodium citrate pH 6.8, 30% PEG 8000 and 15% PEG 400) and flash-frozen in liquid nitrogen. Data collection was carried out at 100 K on beamline ID-30 at ESRF, Grenoble, France. Data collection statistics are summarized in Table S4. The crystals of the apo-form diffracted to a maximum resolution of 1.80 Å, whereas those of the holo-form reached resolutions up to 1.85 Å. The crystals of both the apo- and the holo-form belonged to space group P 1 21 1, the crystal parameters are given in Table S4. The data sets were processed with the program XDS^53^. Initial phases for the holo-enzyme, of which structure was solved first, were obtained by molecular replacement (MR) using the crystal structure of walnut tyrosinase (PDB entry 5CE9)^16^ as the search model. For the apo-enzyme, the solved structure of the holo-form was used as MR search model to deduce initial phases. The structure of both the apo- and the holo-enzyme were then solved by the same procedure: After initial phases were derived, Autobuild^54^ from the PHENIX suite (v. dev-3063)^55^ was used to build the model of apo- and holo-*Sl*PPO1. The resulting model was refined until convergence using phenix. refine^56^, whereby the models were further improved by manual building using COOT (v. 0.8.9.1)^57^. The quality of the final models was verified and evaluated by the MolProbity server before deposition in the PDB (PDB entry of apo-*Sl*PPO1 = 6HQJ and of holo-*Sl*PPO1 = 6HQI). Since the apo- and holo-form of *Sl*PPO1 differed significantly in some structural aspects, five further data sets were evaluated in the same way as described above in order to confirm the absence or presence of specific structural features (Table S2).

### Molecular docking

Docking was performed using Autodock Vina^58^ to identify binding poses of monophenolic (tyramine and phloretin) and diphenolic substrates (dopamine and caffeic acid) within the active centre of *Sl*PPO1 (holo-form structure) in order to structurally analyse substrate binding. The crystal structure of *Sl*PPO1 was prepared for molecular docking by adding missing side chains using COOT and the removal of the C-terminal domain (cleavage after Pro346) in order to create the active form of the isoenzyme. The gate residue Phe270 was defined as flexible residue and the exhaustiveness was set to 100. Structures of the substrates were obtained from the PDB and formatted into pdbqt files using AutoDockTools (ADT, v. 1.5.6)^58^, which specifies and samples all rotatable bonds and computes partial charges for the substrate structures. Binding poses were searched in a grid box of 12 × 12 × 12 Å^3^ (spacing = 1.0 Å) centred in between the two copper ions of the active site. The docking settings (i.e. the grid box) were tested with the structure of TYR from *Bacillus megaterium* (*Bm*TYR) using tyrosine as substrate. The resulting docking poses obtained from Autodock Vina applying our settings resembled almost perfectly the tyrosine pose found in the crystal structure of the *Bm*TYR-tyrosine complex (PDB entry 4P6R)^1^ indicating that the defined settings were suitable. Docking was performed with all important protonation states of each substrate (Fig. S14). Upon docking the binding poses were evaluated by superimposing the docked substrate position with that of tyrosine from the *Bm*TYR-tyrosine structure. Poses that significantly deviated from the binding pose of tyrosine were flagged as ‘unfavourable’ poses. The same procedure was also performed for *Sl*PPO2 by using a homology model of this enzyme, which was prepared by the SWISS-MODEL workspace^59^ based on the structure of *Sl*PPO1. In this case, Phe263 was defined as flexible residue.

## Supplementary information

Supplementary Information
