# Supplementary material for: Biochemical and structural characterization of tomato polyphenol oxidases provide novel insights into their substrate specificity
Source: Sci Rep. 2019 Mar 11;9:4022. doi: 10.1038/s41598-019-39687-0 (PMC6411738; doi:10.1038/s41598-019-39687-0)
Supplement: Supplementary file 1 — Supplementary Information [file 41598_2019_39687_MOESM1_ESM.pdf]

Supporting Information

Biochemical and structural characterization of tomato polyphenol oxidases provide novel insights into their substrate specificity

Ioannis Kampatsikas,[1] Aleksandar Bijelic[1] and Annette Rompel,*[1]

[a] Universität Wien, Fakultät für Chemie, Institut für Biophysikalische Chemie, Althanstraße 14, 1090 Wien, Austria; https://www.bpc.univie.ac.at

Correspondence to: annette.rompel@univie.ac.at

Figures

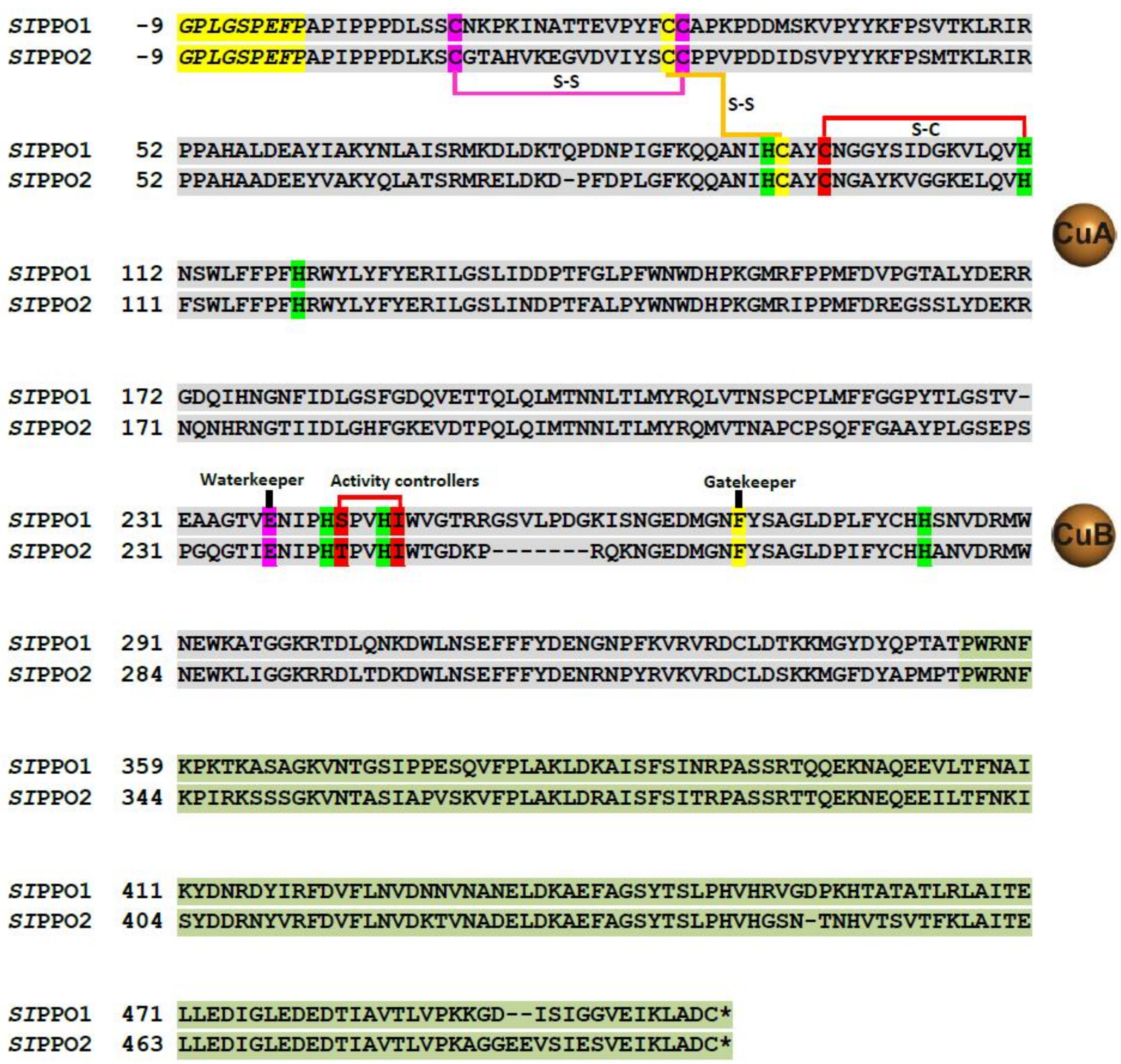

Figure S1. Sequence alignment of the recombinant latent tomato PPOs (*Sl*PPO1 and 2). Highlighted are the conserved copper-coordinating histidines (green) of the dicopper centre, the conserved cysteines that putatively form stabilizing disulfide bonds (S-S), the thioether bridge (Th-br) between a cysteine and the second CuA coordinating histidine (S-C), the conserved glutamic acid: waterkeeper residue (magenta), the conserved phenylalanine: gatekeeper residue (yellow), the two amino acids next to the first and the second conserved histidines of CuB: activity controllers (red). The yellow part of the sequence belong to the expression vector, the grey part of the sequence represents the active domain while the light green part represents the C-terminal domain.

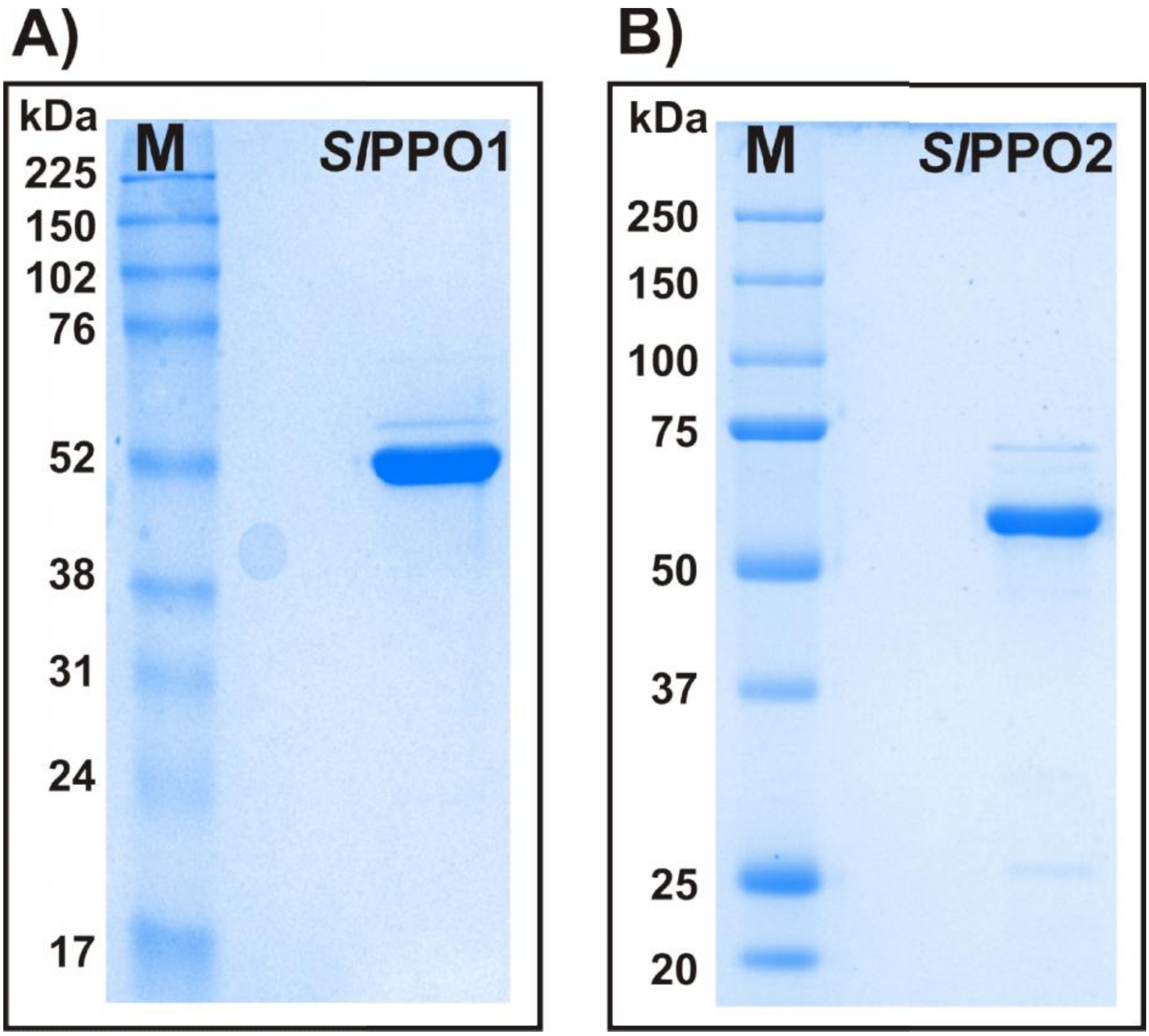

Figure S2. SDS-PAGE of *Sl*PPO1 and *Sl*PPO2. A) *Sl*PPO1 and **B)** *Sl*PPO2 after the last purification step. **(M):** Molecular weight marker. The gels show the full length of the SDS-PAGEs.

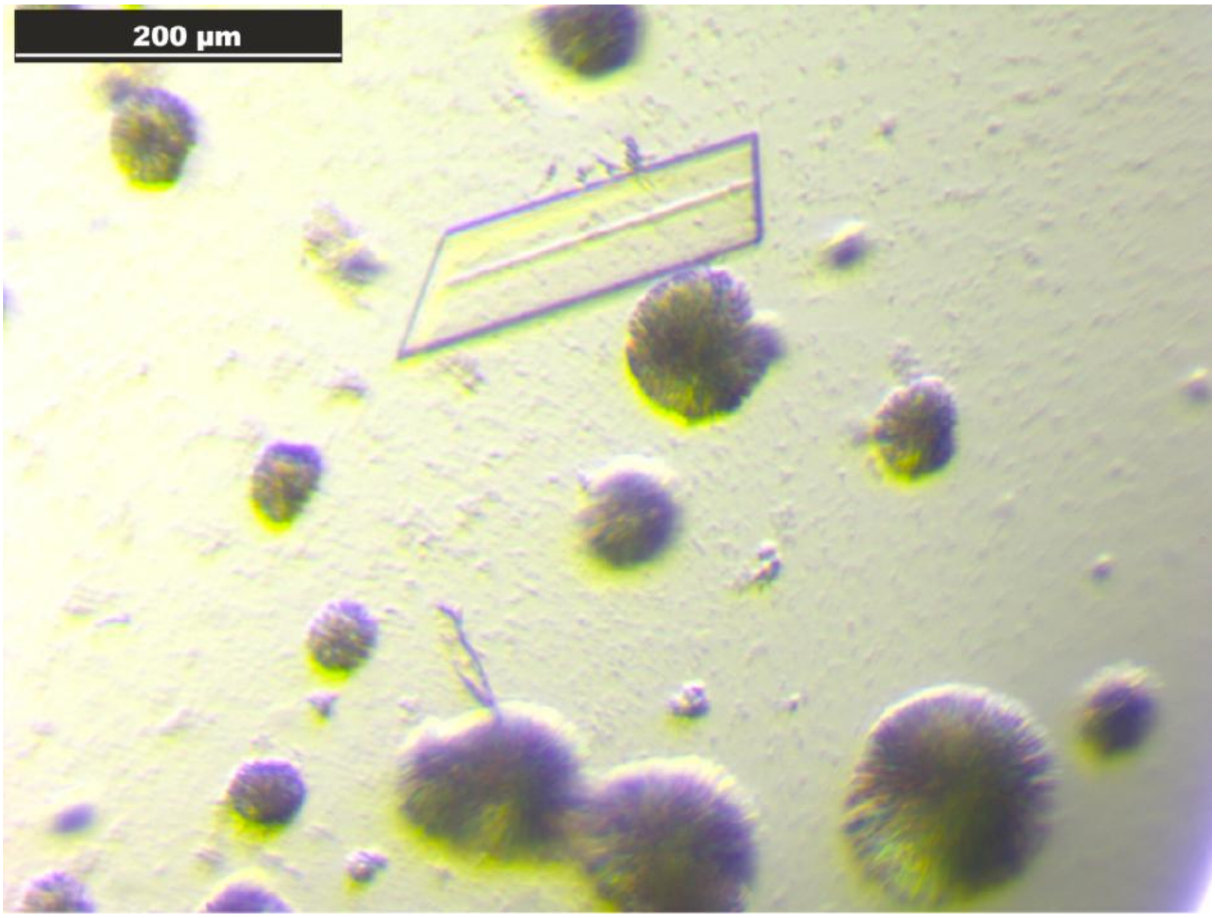

Figure S3. Crystals of *Sl*PPO2 were obtained in 14.5 % PEG3350, 200 mM KNO_3, 200 mM glycine and 75 mM Tris-HCl at pH 7.0.

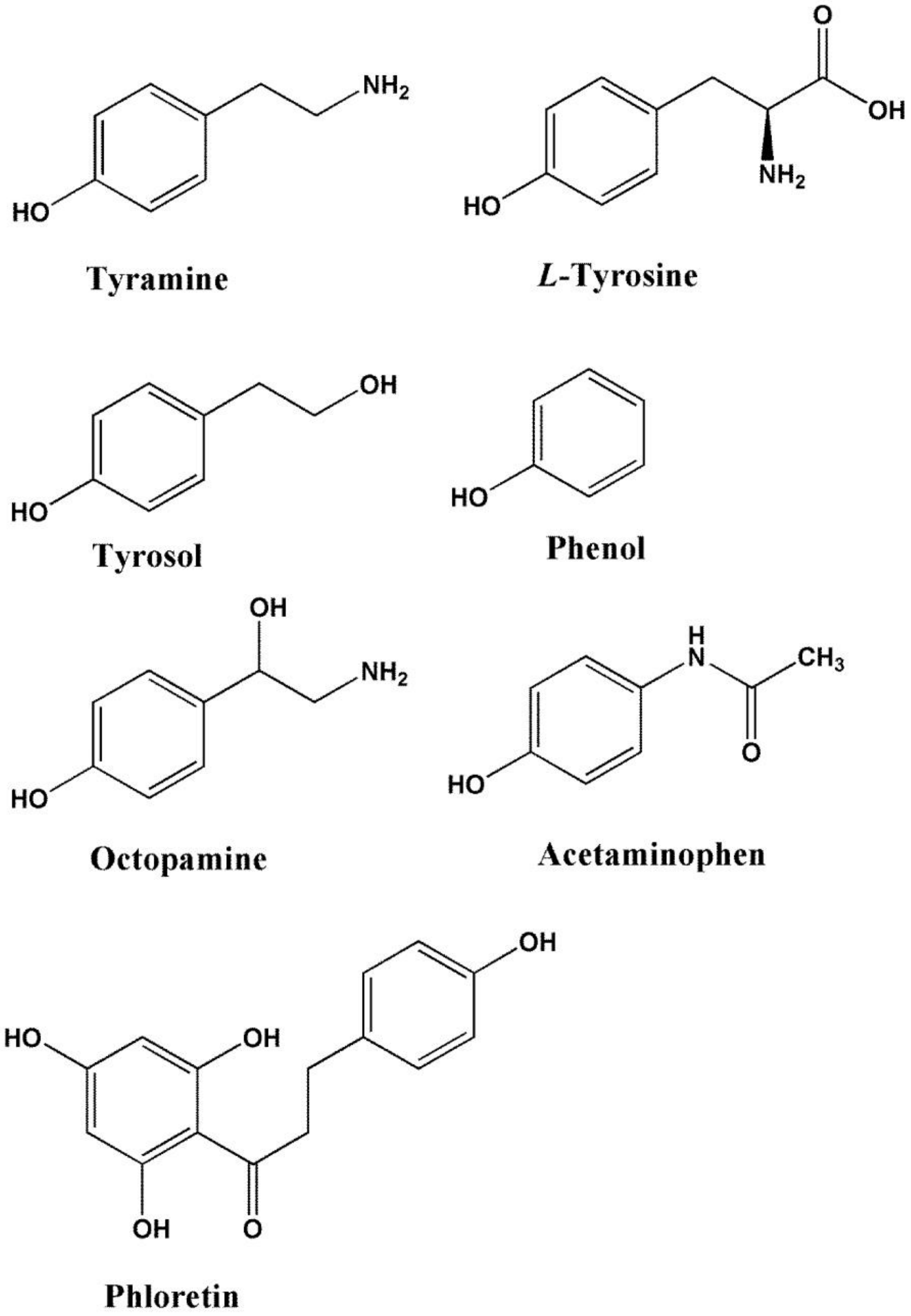

Figure S4. Structural formulae of the tested monophenolic substrates during the kinetic and substrate acceptance assay.

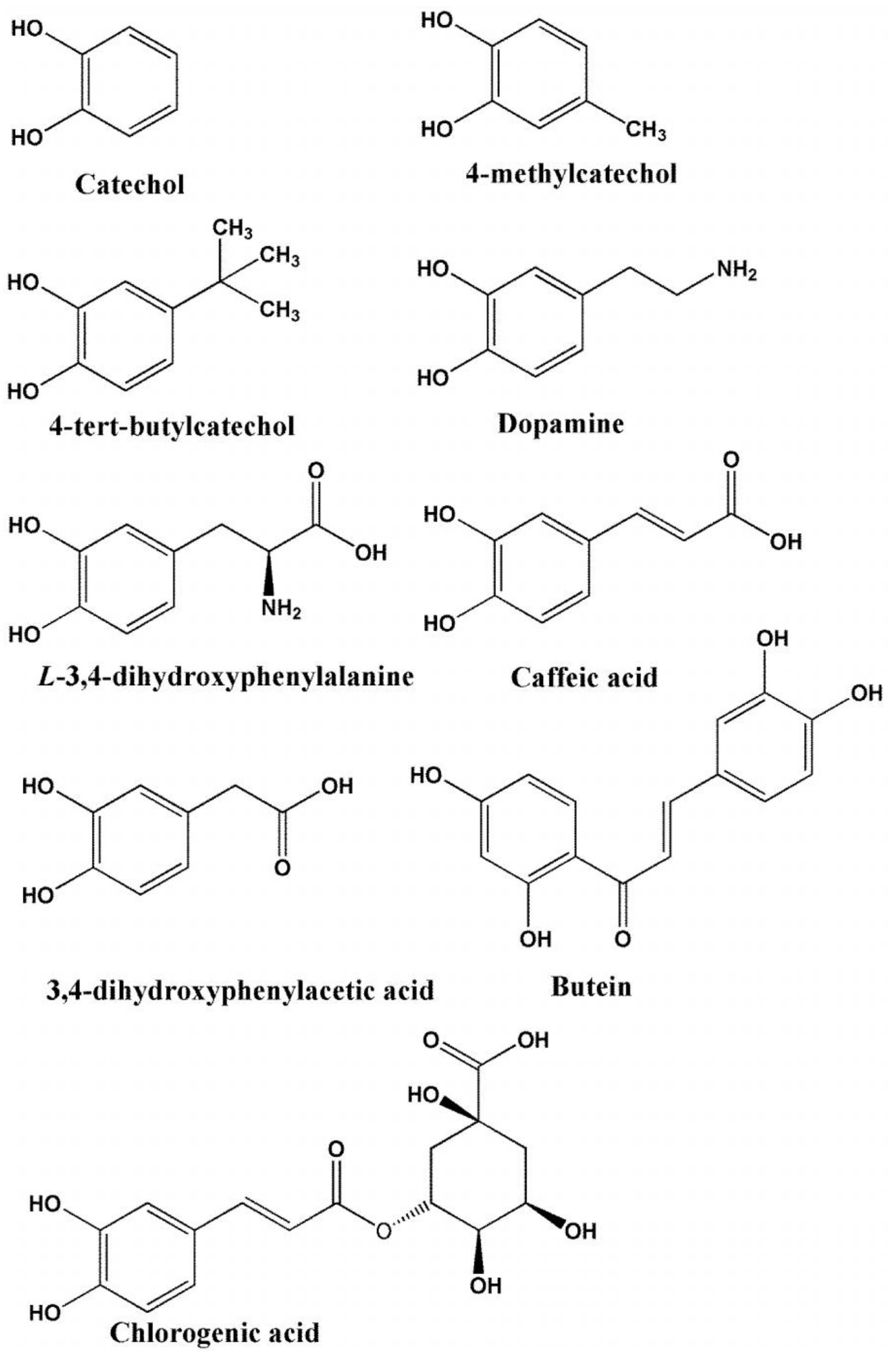

Figure S5. Structural formulae of the tested diphenolic substrates during the kinetic and substrate acceptance assay.

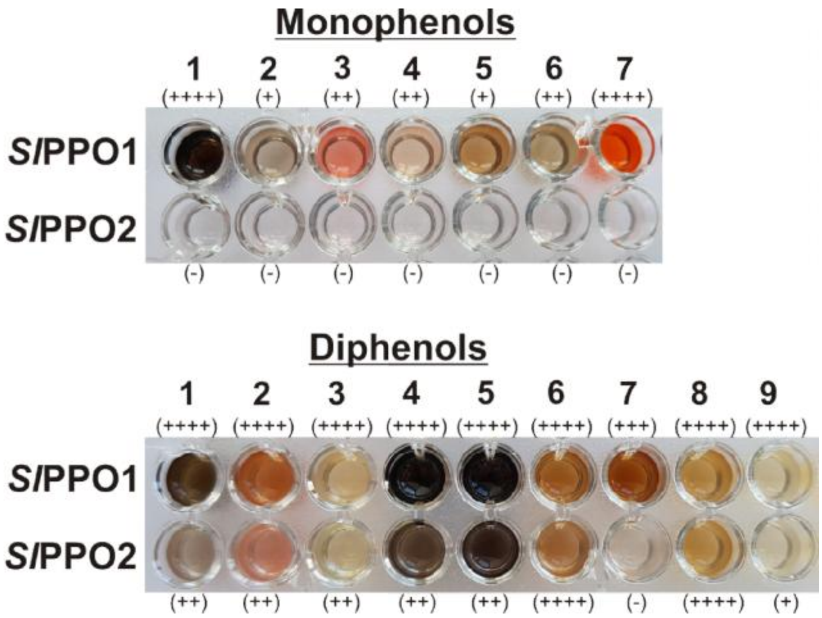

Figure S6. Substrate acceptance assays monitoring the catalytic reactions of *Sl*PPO1 and *Sl*PPO2 with monophenolic and diphenolic substrates. Enzymatic reactions were performed in a total volume of 200 µl containing 50 mM Tris-HCl buffer pH 7.0, 20 µg enzyme for monophenolic and 2 µg enzyme for diphenolic substrates. **Monophenols:** 1) 2 mM tyramine, 2) 1.5 mM tyrosine, 3) 2 mM tyrosol, 4) 2 mM (±)-octopamine, 5) 2 mM phenol, 6) 2 mM acetaminophen and 7) 2 mM phloretin. **Diphenols:** 1) 2 mM catechol, 2) 2 mM 4-methylcatechol, 3) 2 mM 4-tertbutylcatechol, 4) 2 mM dopamine, 5) 2 mM *L*-3,4-dihydroxyphenylalanine (*L*-DOPA), 6) 2 mM caffeic acid, 7) 2 mM 3,4-dihydroxyphenylacetic acid (DOPAC), 8) 2 mM chlorogenic acid and 9) 0.2 mM butein. The activity was characterized and ranked according to the velocity of chromophore formation: (++++) = very fast (1 - 60 seconds), (+++) = fast (60 - 300 seconds), (++) = slow (300 - 1500 seconds), (+) = very slow (1500 seconds - 12 hours) and (-) = inactive.

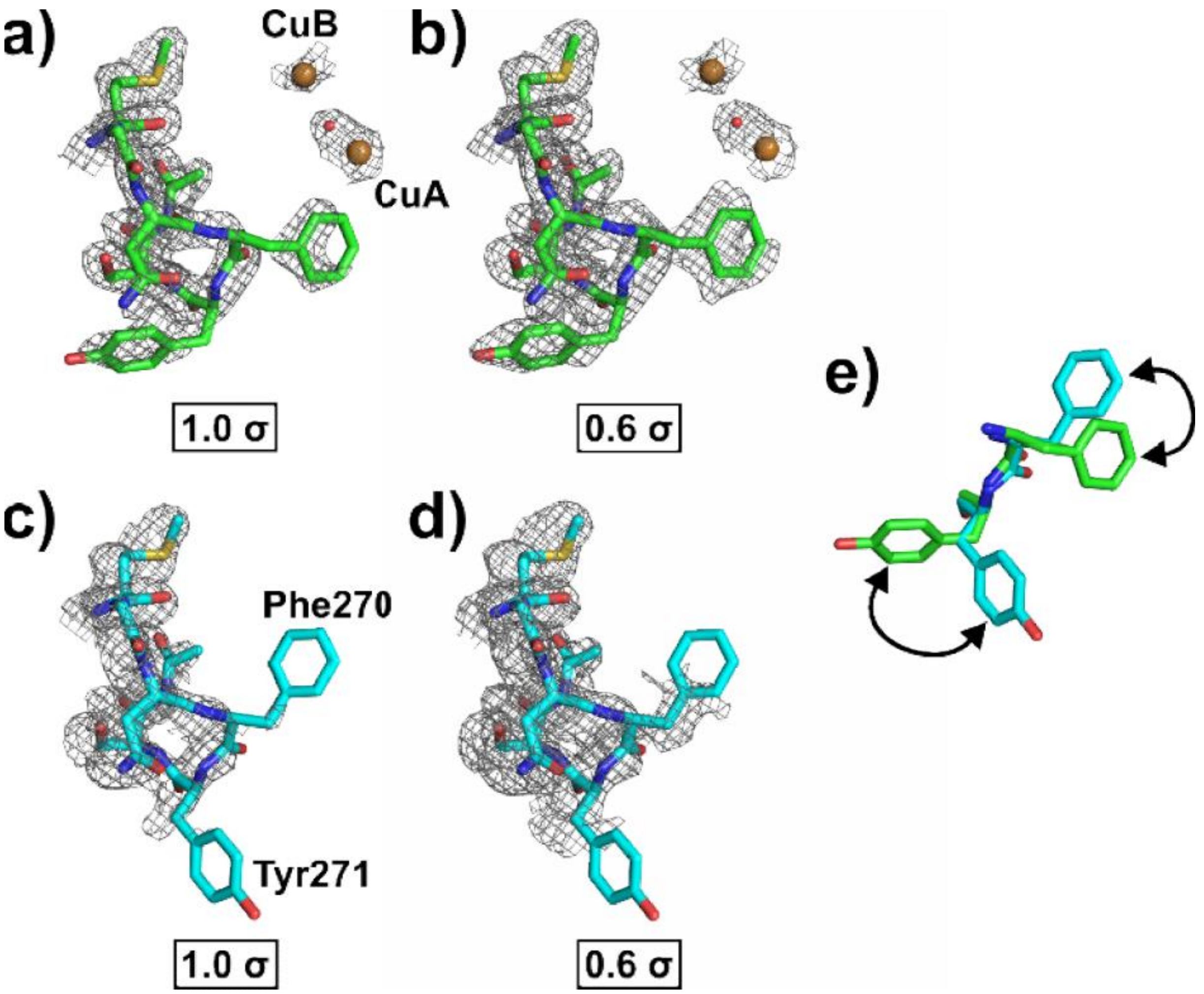

Figure S7. Position of the gatekeeper residue Phe270 in holo-*Sl*PPO1 (PDB: 6HQI) and apo-*Sl*PPO1 (PDB: 6HQJ). The 2mF$_o$-DF$_c$ maps (grey mesh) of holo- (green structure) and apo-*Sl*PPO1 (cyan structure) are shown. **a)** Portion of the 2mF$_o$-DF$_c$ map of holo-*Sl*PPO1 contoured at 1.0 σ. Slight electron density for the putative main position of Phe270 is visible. **b)** Same portion and map as shown in a) but contoured at 0.6 σ. Only at this contour level clear electron density for Phe270 starts to appear. **c)** Portion of the 2mF$_o$-DF$_c$ map of apo-*Sl*PPO1 contoured at 1.0 σ. No electron density for the putative main position of Phe270 is observable at this contour level. **d)** Same portion and map as shown in c) but contoured at 0.6 σ. Only at this contour level slight electron density starts to appear for Phe270. **e)** Superimposition of the peptide Phe270-Tyr271 of holo- and apo-*Sl*PPO1. The significant difference regarding the position of both the gatekeeper residue Phe270 and the adjacent Tyr271 between the holo- and apo-form of *Sl*PPO1 is visible.

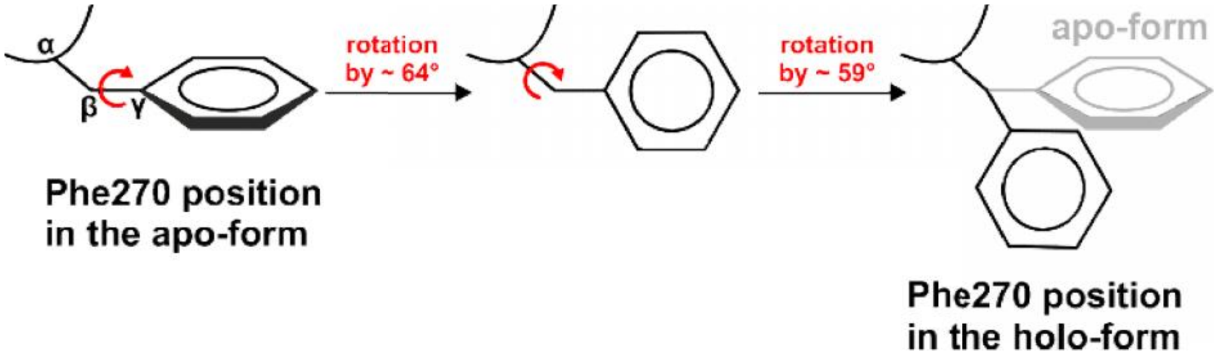

Figure S8. Differences in the Phe270 position between the holo- and the apo-form. The gatekeeper residue in the holo-form is significantly shifted in comparison to that of the apo-structure. A rotation around the Cβ-Cγ bond of Phe270 in the apo-form by ~64° followed by a second rotation around the Cα-Cβ bond by ~ 59° is required to superimpose it with the corresponding position of Phe270 in the holo-form.

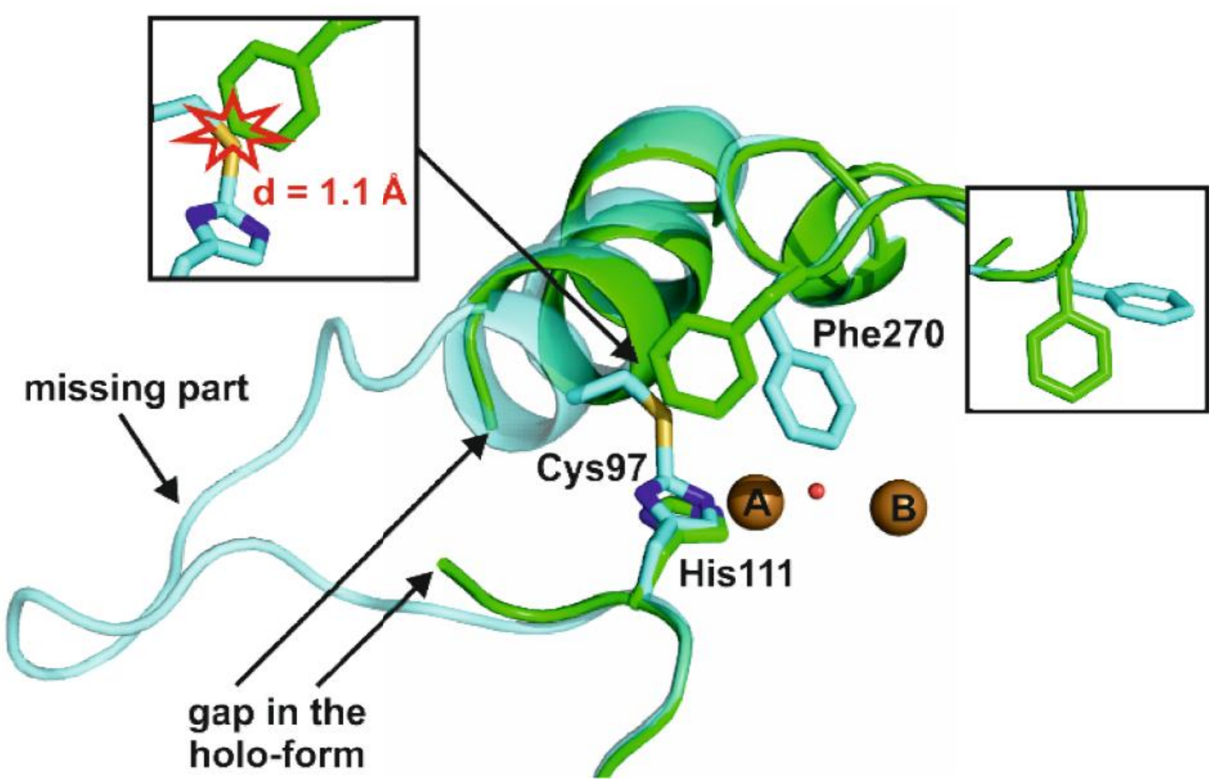

Figure S9. Structural differences between the holo- and the apo-form. The structural differences between the holo- (green cartoon) and the apo-form (cyan cartoon) are depicted by structural superimposition. The holo-form lacks the Cys97-Leu117 loop (shown as transparent cyan loop) and thus also the thioether bridge forming Cys97, wherefore, the thioether bridge cannot be formed. However, if the thioether bridge was presented in the holo-form (at the same position as in the apo-form), the gatekeeper residue Phe270 (in its current holo-position) would sterically interfere with the bridge (left inset). Thus, it seems that the thioether bridge might stabilize the position of the gatekeeper residue. The right inset shows once again the different positioning of Phe270 in the holo- and apo-form.

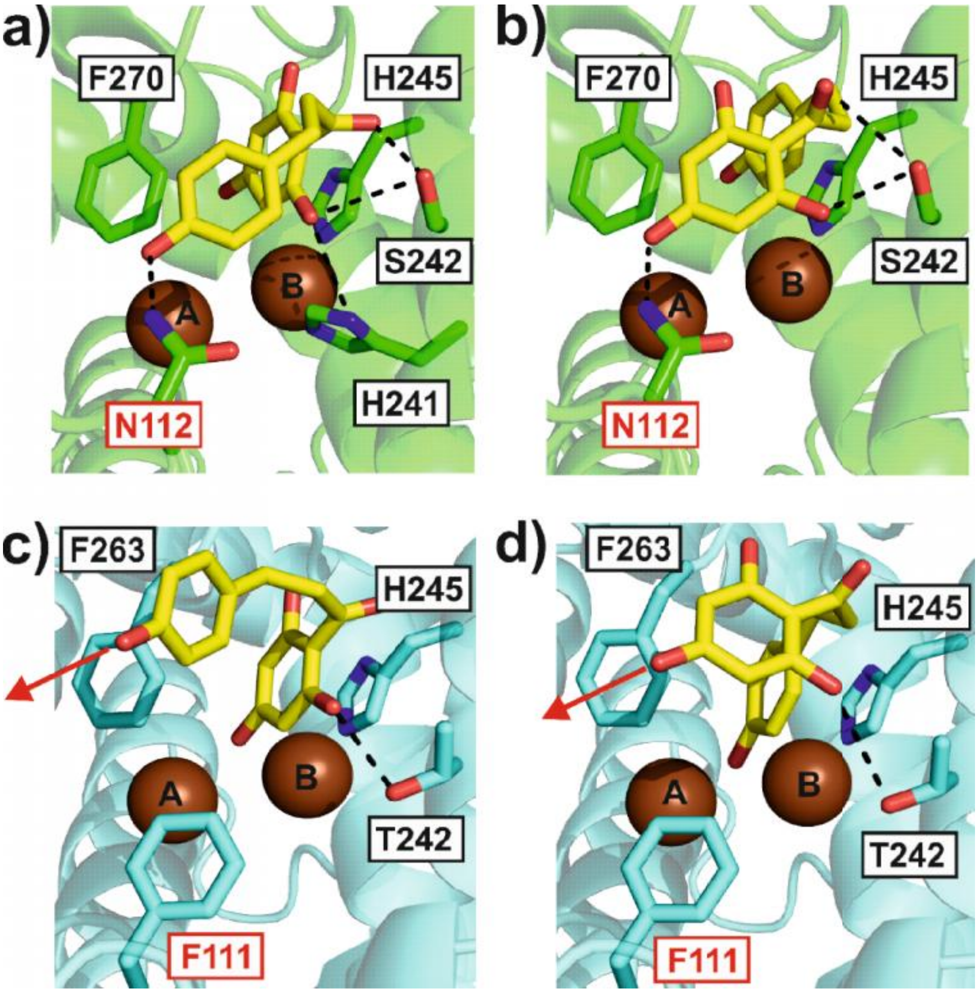

Figure S10. Structural comparison of the docking poses of the *Sl*PPO1-phloretin and *Sl*PPO2-phloretin complexes. a) *Sl*PPO1-phloretin complex exhibiting binding pose 1 (2,4,6-trihydroxyphenyl ring pointing towards the active site). **b)** *Sl*PPO1-phloretin complex exhibiting binding pose 2 (2,4,6-trihydroxyphenyl ring pointing away from the active site). **c)** *Sl*PPO2-phloretin complex exhibiting binding pose 1 (2,4,6-trihydroxyphenyl ring pointing towards the active site). **d)** *Sl*PPO2-phloretin complex exhibiting binding pose 2 (2,4,6-trihydroxyphenyl ring pointing away from the active site). Both enzymes are able to bind both rings of phloretin. However, the figures indicate that there is one significant structural difference between the active sites of *Sl*PPO1 and *Sl*PPO2, showing that *Sl*PPO1 possesses an asparagine at position 112 (N112, indicated in red), whereas *Sl*PPO2 contains a phenylalanine (F111, indicated in red) at the corresponding position. This difference leads to the fact that the *Sl*PPO2-phloretin interactions are significantly less pronounced than in the corresponding *Sl*PPO1-phloretin complex. In both poses (Figure c and d) *Sl*PPO2 interacts with the substrate only by its activity controller threonine (T242) *via* H-bonding with an *ortho*-positioned hydroxyl group of the 2,4,6-trihydroxyphenyl moiety. In contrast, *Sl*PPO1 does not only form H-bonds with its activity controller serine (S242) but in addition exhibits strong interactions with the aforementioned asparagine (N112, Figure a and b). This interactions is lacking in the *Sl*PPO2-phloretin complex as the bulky and hydrophobic phenylalanine (F111) is not able to stabilize the second ring (pointing away from the active site) of phloretin. Moreover, it seems that the bulky F111 is rather pushing away the second ring of phloretin out of the active site cleft into the bulk solvent (indicated by red arrows in Figure c and d),

which might lead to the destabilization of the whole complex. This could partially explain the lack of binding between phloretin and *Sl*PPO2 (as evidenced by kinetics).

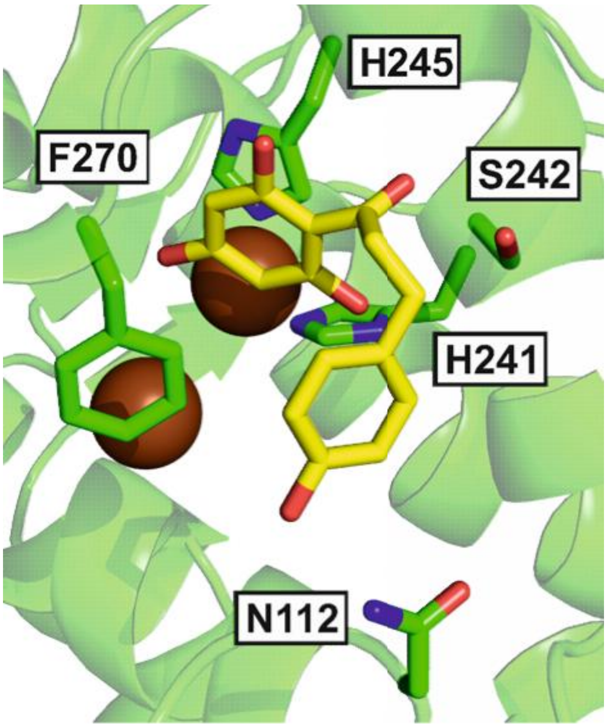

Figure S11. Binding of phloretin to *Sl*PPO1 (binding pose 1). The figure shows the binding of phloretin to *Sl*PPO1 (binding pose 1) from another perspective (in comparison to Figure 5) in order to better understand the conformation of the substrate within the enzyme's active site.

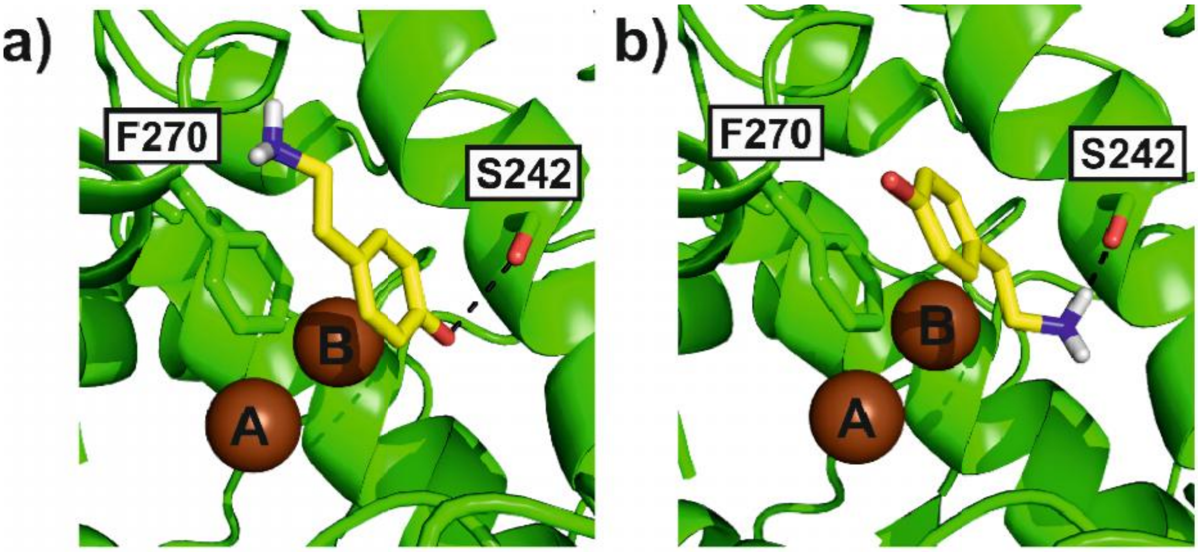

Figure S12. Gatekeeper residue directed substrate poses blocking substrate entrance into the active site. **a)** The gatekeeper residue Phe270 of *Sl*PPO1 interacts (*via* π-stacking) with the small monophenolic substrate tyramine in such a way that the substrate is blocked away from the dicopper centre (in this positioning Phe270 is covering CuA). This unfavourable substrate pose is additionally stabilized by a H-bond between the hydroxyl group of the monophenol with the activity controller Ser242. **b)** Similar scenario as depicted in a), the orientation of the substrate is just different. Again, tyramine is prevented from entering the active site by Phe270-mediated unfavourable π-interactions. In addition, the amino group of tyramine forms a H-bond with Ser242, which contributes to the 'stucking' of the substrate in this unfavourable pose.

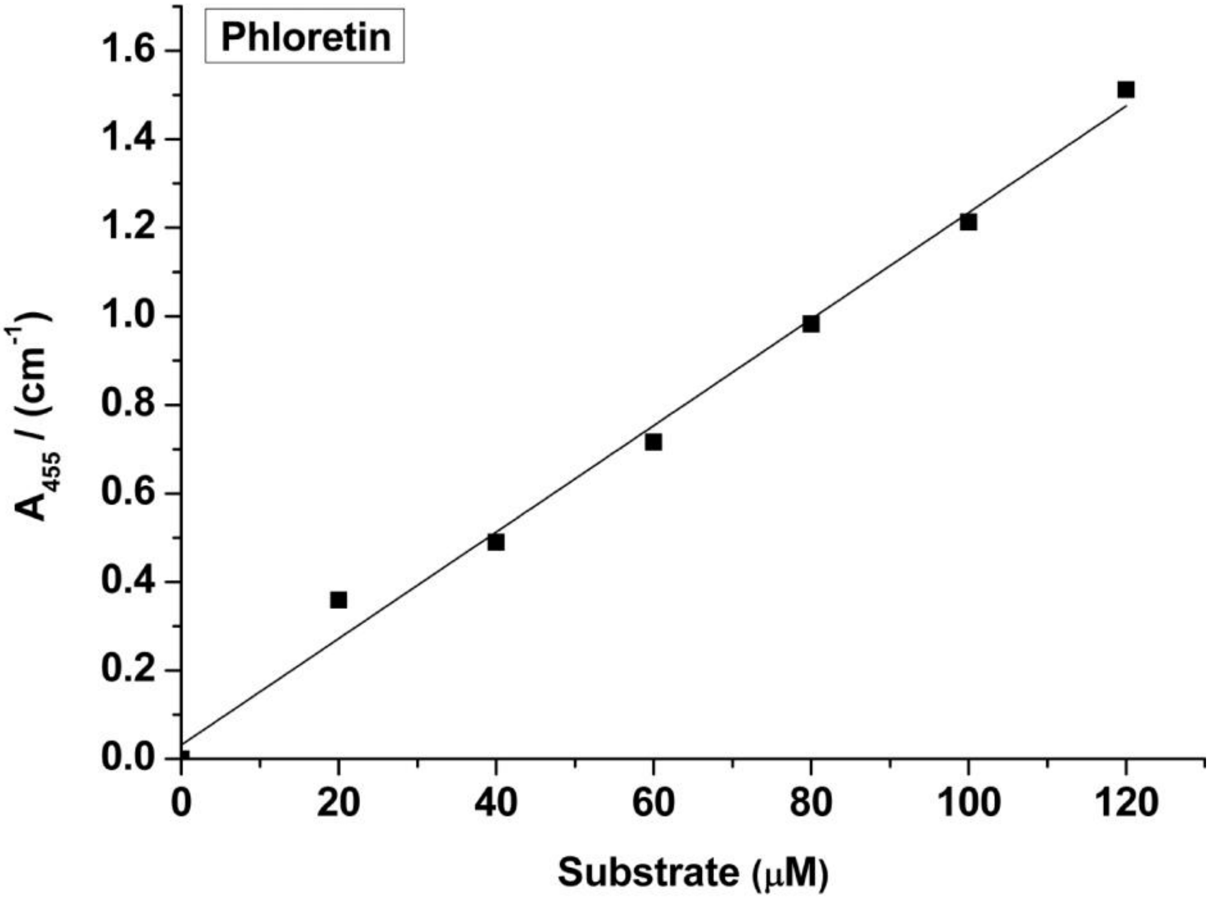

Figure S13. Absorbances obtained by hydroxylation of the substrate phloretin at 455 nm with 30 µg of latent *Md*PPO1 and 3 mM SDS as activator (see Methods).

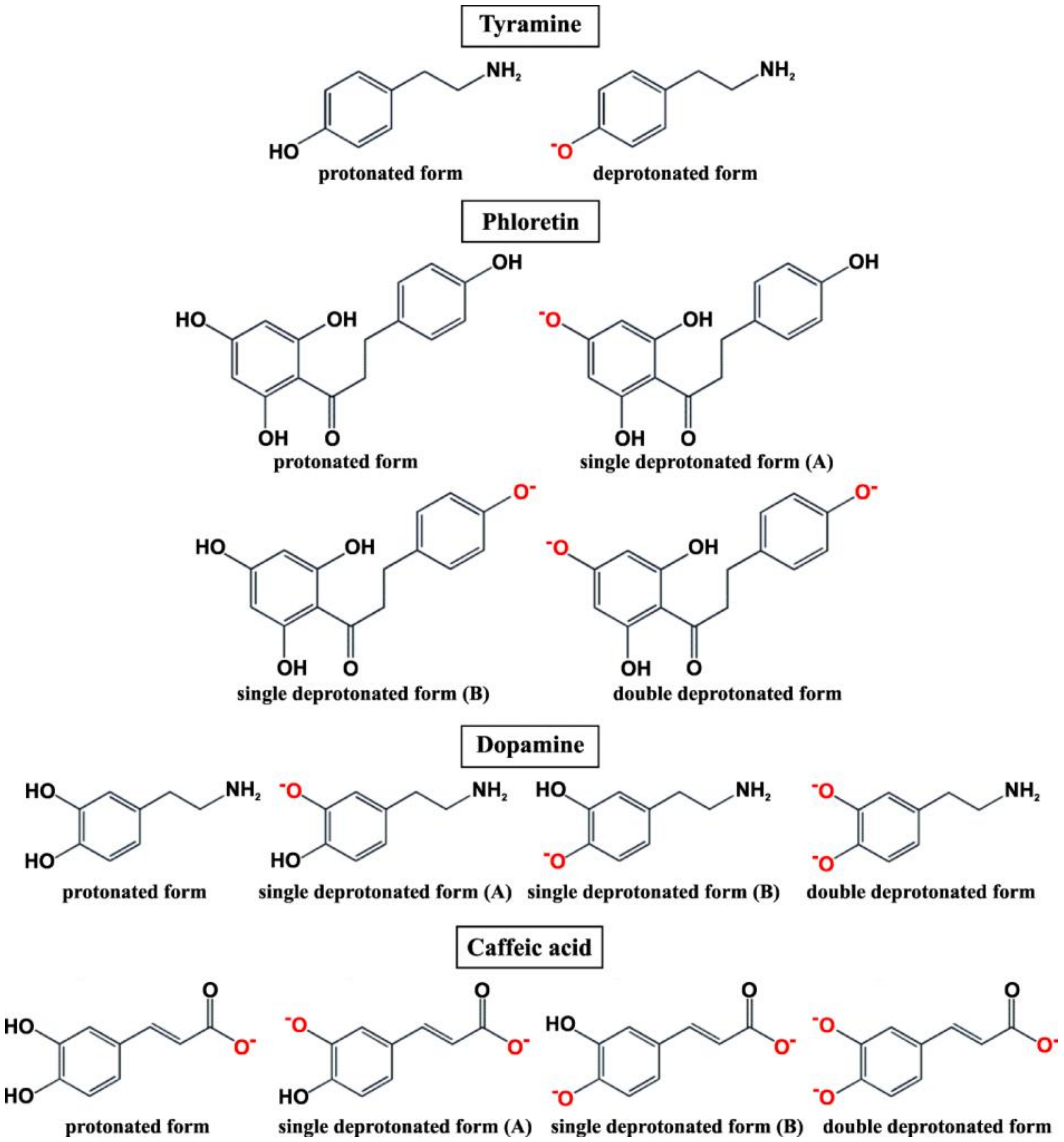

Figure S14. Protonation states of all substrates. The figure depicts all protonation states of each substrate that were used for the docking experiment.

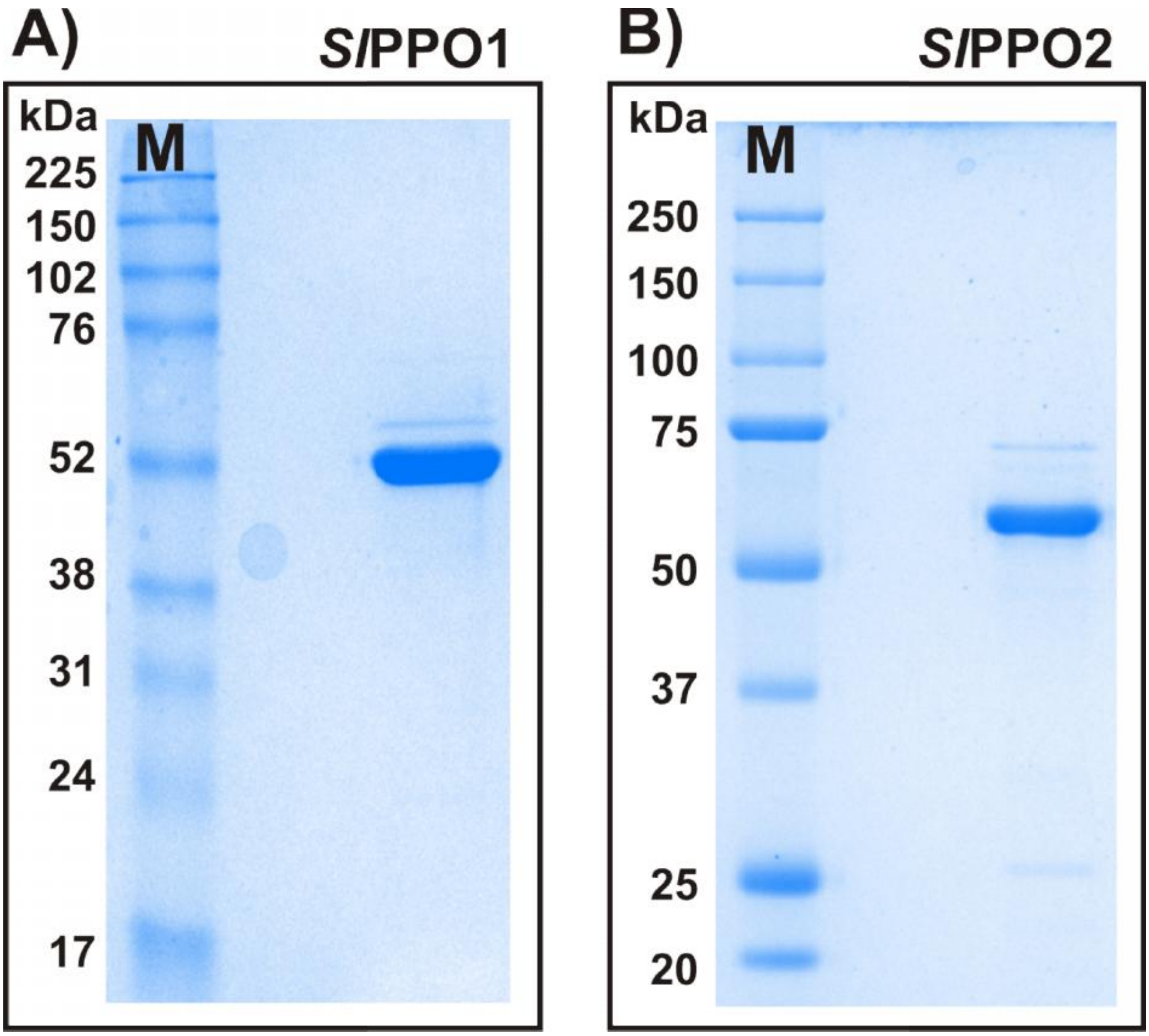

Figure S15. Full-length gels of Figure S2.

Tables

Table S1: Data base sequences and primers used to amplify the expressed genes.

Primers	PPO	Accession number	Size [bp]	ORF [AA]*	MW [kDa]
fw 5´ ARCWCCTAYWCCAYCHCCT 3´<br>rev 5´ TCAAACATGGAAATTAAAAAACAAACAG 3´	*Sl*PPO1	LR025217	1521	506	57.14
fw 5´ ARCWCCTAYWCCAYCHCCT 3´<br>rev 5´ TTAACAATCMDCAAGMYTGAT 3´	*Sl*PPO2	LS999938	1503	500	57.05

Table S2. Comparison of several apo-and holo structures regarding specific structural features.

structure	Conserved water	Thioether bridge	copper	Phe270 density
holo-form 1*	no	no	yes	very low
holo-form 2	yes	no	yes	very low
holo-form 3	no	no	yes	very low
holo-form 4	no	no	yes	very low
holo-form 5	yes	no	CuA only	no
apo-form 1*	yes	yes	no	very low
apo-form 2	yes	yes	no	no

* This structure is discussed in the main text and was deposited in the PDB.

Table S3. Affinity scores of the docking experiment computed by Autodock Vina.

Substrate	Affinity (kcal/mol)	State of the docked substrate	K_m / (mM)
tyramine	- 4.7	OH-group deprotonated	0.69 ± 0.12
phloretin	- 7.2	*para*-OH group of the trihydroxyphenyl moiety deprotonated	0.11 ± 0.01
dopamine	- 4.2	both OH-groups deprotonated	0.67 ± 0.13
caffeic acid	- 6.6	both OH-groups deprotonated	0.72 ± 0.04

Table S4. Data collection and processing.

Protein	holo-*Sl*PPO1 (PDB 6HQI)	apo-*Sl*PPO1 (PDB 6HQJ)
Diffraction source	ID-30, ESRF	ID-30, ESRF
Wavelength (Å)	0.9677	0.9660
Temperature (K)	100	100
Detector	PILATUS3 2M	PILATUS3 2M
Rotation range per image (°)	0.10	0.15
Total rotation range (°)	360.0	167.1
Exposure time per image (s)	0.02	0.21
Space group	$P12_11$	$P12_11$
a, *b*, *c* (Å)	60.62, 54.10, 69.82	60.80, 54.14, 69.77
α, β, γ (°)	90.00, 104.60, 90.00	90.00, 101.08, 90.00
Mosaicity (°)	0.296	0.547
Resolution range (Å)	42.23 - 1.85 (1.92 - 1.85)	42.47 - 1.80 (1.87 - 1.80)
Total No. of reflections	253455 (24698)	124679 (11482)
No. of unique reflections	37575 (3726)	40854 (3972)
Completeness (%)	99.95 (99.97)	98.95 (97.66)
Redundancy	6.7 (6.6)	3.1 (2.9)
$\langle I/\sigma(I)\rangle$	6.81 (1.17)	10.50 (1.68)
$R_{p.i.m}$[a]	0.072 (0.590)	0.049 (0.441)
$CC_{1/2}$[b]	0.992 (0.551)	0.997 (0.665)
Overall *B* factor from Wilson plot ($Å^2$)	26.63	21.07

[a] $R_{p.i.m.} = \Sigma_{hkl}[n/(n-1)]^{1/2}\ \Sigma_i|I_i(hkl) - \langle I(hkl)\rangle| / \Sigma_{hkl}\ \Sigma_i\ I_i(hkl)$, where n is the multiplicity, $I_i(hkl)$ is the ith observation of reflection hkl and <I(hkl)> is the weighted average intensity for all observations of reflection hkl. [b] The mean intensity correlation coefficient of half-datasets. Values in parentheses are for the highest resolution shell.
